# PIXiE: an algorithm for automated ion mobility arrival time extraction and collision cross section calculation using global data association

**DOI:** 10.1093/bioinformatics/btx305

**Published:** 2017-05-15

**Authors:** Jian Ma, Cameron P Casey, Xueyun Zheng, Yehia M Ibrahim, Christopher S Wilkins, Ryan S Renslow, Dennis G Thomas, Samuel H Payne, Matthew E Monroe, Richard D Smith, Justin G Teeguarden, Erin S Baker, Thomas O Metz

**Affiliations:** 1 Biological Sciences Division, Pacific Northwest National Laboratory, Richland, WA, USA; 2 Department of Environmental and Molecular Toxicology, Oregon State University, Corvallis, OR, USA

## Abstract

**Motivation:**

Drift tube ion mobility spectrometry coupled with mass spectrometry (DTIMS-MS) is increasingly implemented in high throughput omics workflows, and new informatics approaches are necessary for processing the associated data. To automatically extract arrival times for molecules measured by DTIMS at multiple electric fields and compute their associated collisional cross sections (CCS), we created the PNNL Ion Mobility Cross Section Extractor (PIXiE). The primary application presented for this algorithm is the extraction of data that can then be used to create a reference library of experimental CCS values for use in high throughput omics analyses.

**Results:**

We demonstrate the utility of this approach by automatically extracting arrival times and calculating the associated CCSs for a set of endogenous metabolites and xenobiotics. The PIXiE-generated CCS values were within error of those calculated using commercially available instrument vendor software.

**Availability and implementation:**

PIXiE is an open-source tool, freely available on Github. The documentation, source code of the software, and a GUI can be found at https://github.com/PNNL-Comp-Mass-Spec/PIXiE and the source code of the backend workflow library used by PIXiE can be found at https://github.com/PNNL-Comp-Mass-Spec/IMS-Informed-Library.

**Supplementary information:**

Supplementary data are available at *Bioinformatics* online.

## 1 Introduction

The field of metabolomics has made great strides since its early explorations by Pauling and Robinson (Pauling *et al.* (1971), Robinson *et al.* (1973), Robinson *et al.* (1974)), and conceptualization as an ‘omics’ by Nicholson *et al.* (1999). Many analytical platforms exist for collecting metabolomics data, although methods based upon gas chromatography (GC) and liquid chromatography (LC) coupled with mass spectrometry (MS) dominate most applications. Similarly, many algorithms and software packages are available for analyzing the data in the associated informatics pipelines. These informatics tools are used for identification and alignment of detected features (i.e. initially unidentified, putative metabolites) across multiple analyses (LaMarche *et al.* 2013; Pluskal *et al.* 2010; Smith *et al.* 2006), normalization of metabolite abundance information (Polpitiya *et al.* 2008; Xia *et al.* 2015), and matching the experimental metabolite characteristics to reference libraries containing spectra, retention times or chemical shifts from analyses of authentic chemical standards ( Gerlich and Neumann 2013, Kind *et al.* 2013; Weljie *et al.* 2006). However, a desire for the complete characterization of both xenobiotics and endogenous chemicals in human exposures has initiated a transformation in metabolomics (Council 2012; Metz *et al.* 2016; Soltow *et al.* 2011), resulting in the need for measurements that provide much higher throughput while still maintaining high sensitivity.

Drift tube ion mobility spectrometry (DTIMS) is a rapid gas phase separation technique that is easily combined with MS for high throughput multi-dimensional separations (Guevremont *et al.* 1997; Steiner *et al.* 2001). In DTIMS, ions are subject to a constant electric field while traveling through a buffer gas and separate quickly based on ion shape and size, e.g. compact species drift faster than those with extended structures (Guevremont *et al.* 1997; Mason and McDaniel 1988). By measuring DTIMS arrival times it is possible to derive a molecule’s collisional cross section (CCS), which characterizes its chemical structure and increases the specificity of metabolite identifications, particularly when combined with accurate mass measurements. Herein, we report an approach for automatically extracting arrival times for metabolites from DTIMS-MS measurements and for subsequently calculating their CCS values, where multiple electric fields are utilized to obtain the most accurate experimental CCS measurements. Given a list of empirical formulae for target molecules of interest, our algorithm analyzes the DTIMS-MS data for each target ion and extracts the arrival times at the peak centroids for each electric field measurement. These arrival times are then utilized to calculate the CCS for each ion. When candidate ions are indistinguishable using mass information alone, the algorithm calculates and tracks CCSs for all possible candidate ions throughout the experiment by implementing a unique, global data association approach inspired from multi-object tracking in video sequences (Berclaz Fleuret *et al.* 2011; Siems *et al.* 1994). The resulting algorithm, implemented in the PNNL Ion Mobility Cross Section Extractor (PIXiE) software supports the extraction of arrival times, molecular CCSs and accurate mass data for the rapid construction of reference libraries for use in high throughput DTIMS-MS-based studies.

## 2 Materials and methods

### 2.1 Overview: CCS calculations from IMS data

Reference libraries for high throughput identification of molecules ideally contain two or more orthogonal metrics such as accurate mass, isotopic signature, LC elution time, NMR spectra, MS/MS spectra, or DTIMS CCS values (Lapthorn *et al.* 2013). These criteria adhere to the recommendations of the Metabolomics Society’s Metabolomics Standards Initiative for highest confidence in metabolite identification (Castle *et al.* 2006; Sumner *et al.* 2007). While methods for experimentally determining molecular CCS values are well established, software implementations to calculate these values remain limited with currently available open source tools either needing calibrant ions to calculate CCS values, requiring that CCS values be provided, or having difficulties calculating values for molecules other than native proteins (Allison *et al.* 2015; Eschweiler *et al.* 2015).This manuscript describes the automatic extraction of measured drift times and calculation of associated experimental CCS values by collecting ion arrival time distributions (ATDs) from analyses conducted at multiple, different electric fields (seven in this demonstration), a capability which is not currently available for DTIMS in open source format. Although it is possible to determine molecular CCS values experimentally using single electric field approaches together with calibration against molecules with known CCS values, we chose to measure CCS directly using multiple DTIMS electric fields, for higher accuracy. While we utilized seven electric fields for each measurement, any number >2 can be used, with higher numbers enabling more accurate results. To acquire CCS, the mobility of each ion must be calculated by extracting the arrival time, *t*_A_, from its peak apex, and then plotting it against *p*/(*TV*), as shown in Equation (1).

(1) $$t_{A}=\frac{l^{2}}{K_{o}}\cdot \frac{273.15}{760}\cdot \frac{p}{V\cdot T}+t_{o}.$$

The expression for *t*_A_ is an equation for a straight line (*y* = *mx* + *b*) where the slope of the line is inversely proportional to the reduced mobility of the ion (*K_o_*), and the *y*-intercept is equal to the time outside the IMS drift cell (*t_o_*). *t*_A_ is also a function of drift voltage (*V*, in volts), temperature (*T*, in Kelvins), pressure (*p*, in torr) and the physical length of the drift tube (*l*, in meters). Plots of *t*_A_ versus *p*/(*TV*) are highly linear with *R*^2^ values of at least 0.9999. The relationship between the mobility of an ion, *K_o_*, and its CCS has been derived in detail using kinetic theory (Mason *et al.* 1958) and is given by Equation (2).

(2) $$K_{o}=\frac{3q}{16N}\cdot {\left(\frac{2\pi }{\mu k_{B}T}\right)}^{1/2}\cdot \frac{1}{\Omega },$$

where *q* is the ion charge, *N* is the buffer gas density, μ is the reduced mass of the collision partners, *k*_B_ is Boltzmann’s constant and Ω is the momentum transfer collision integral, which describes the collision between the ion and the buffer gas atoms and gives direct information about the conformation of the ion traveling through the drift cell. To reduce errors in the experimental evaluation of Ω, multiple measurements are made on each system studied so that standard deviations can be determined.

**Fig. 1.**
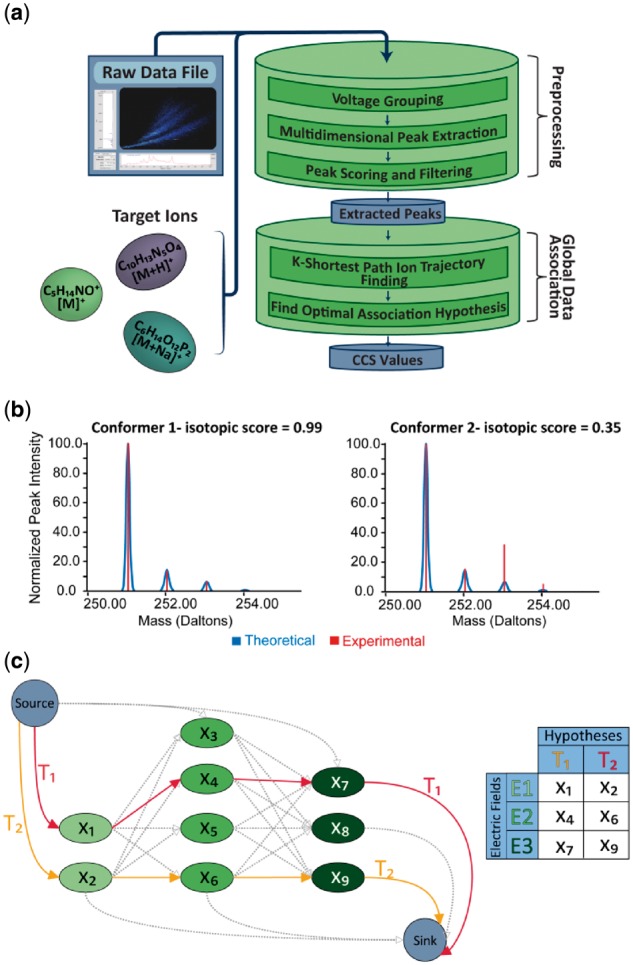
(**a**) Steps of the mobility extraction algorithm. A raw data file in UIMF format and a set of target ions are provided as inputs to PIXiE. The preprocessing step starts after voltage grouping to extract and filter peaks. After filtering, the algorithm uses K-shortest path and maximum *a posteriori* probability estimation to solve the global data association problem and process the peaks into mobility information of conformer ions; (**b**) Application of isotopic score for improved chemical identification. In the example shown, the software extracted two candidate conformers for bisphenol S (sulfonyl diphenol) when given a low isotopic score threshold in the preprocessing step. When the isotopic score threshold is raised, the latter conformer is removed due to its low isotopic score; (**c**) the ion transition graph G. Each path from source to sink represents a potential association hypothesis of ion peaks (*x*_1_…*x_n_*) across multiple electric fields. These hypotheses are evaluated for robustness, and those that pass all filtering steps are used to calculate CCS values. In the example above, two hypotheses, *T*_1_ = (*x*_1_, *x*_4_, *x*_7_) and *T*_2_ = (*x*_2_, *x*_6_, *x*_9_), represent two conformers measured across three electric fields

Automated processing of DTIMS-MS data is required to support high-throughput creation of reference libraries, which would otherwise involve extensive manual data analysis. However, there are challenges associated with automated extraction of CCS as the DTIMS ATD peak at the expected *m/z* of a target ion can correspond to three distinct possibilities: (1) the actual target ion; (2) a co-drifting, unresolved compound that is indistinguishable from the target ion using *m/z*; or (3) noise. Further, to determine the link between observed and expected ions, as well as to identify and remove noise, peaks need to be detected and tracked across multiple electrical fields. We formulated this last challenge as a global data association problem, and refer to each of the potential solutions as an association hypothesis. To solve the global association problem, PIXiE creates the association hypotheses set based on analyses of peak diffusion profiles across electric fields. From this set, PIXiE selects the optimal association hypothesis as the final solution to the global data association problem. The following sections describe the details of each step of the algorithm in the order they are implemented (Fig. 1).

### 2.2 Data collection and target lists

For the CCS calculations made by PIXiE, *n* different electric fields are used. In our case, seven electric fields ranging from 10.8 to 18.5 V/cm were analyzed for 30 s each, resulting in 3.5 min analyses for each ion collected in the same unified ion mobility file (UIMF) (Shah *et al.* 2010). PIXiE requires a target list to begin the analyses. This list consists of known empirical formulae and putative adducts for the molecules of interest in each UIMF. PIXiE then extracts CCSs for all molecular conformers of the target molecules that can be distinguished by the IMS separations.

### 2.3 Peak identification, scoring and filtering

PIXiE performs electric field grouping, multidimensional peak extraction (Crowell *et al.* 2013), scoring, and filtering (Fig. 1). These steps detect candidate peak(s) for each target ion and remove those that do not have sufficient peak quality for further analysis or whose isotopic distribution(s) do not match that of the target empirical formula. These steps are described briefly below.

#### 2.3.1 Electric field grouping and multidimensional peak extraction

The first step in processing the data in PIXiE is to bin the IMS-MS spectra according to the electric field at which the spectrum was collected. Spectra sharing the same electric field are then averaged to enhance the signal-to-noise ratio. DTIMS-MS features are then characterized by *m/z*, arrival time and intensity and extracted from the raw data using a modified version of the LC-IMS-MS Feature Finder (Crowell *et al.* 2013). The original implementation of the feature finder modeled a peak in two dimensions on an intensity map with LC retention time and IMS arrival time as *x*- and *y*-axes, respectively. We modified this algorithm to extract 2D peaks from an intensity map with *m/z* and arrival time as *x*- and *y*-axes. In order to increase computational performance, the peak detector was applied to the 2D intensity area within a ±250-ppm mass window surrounding the expected *m/z* of the target molecule to determine noise and peaks. Filtering of co-drifting compounds based on measured *m/z* is implicitly done in the global data association step, as ions with a measured mass closer to the expected value produce a higher *a posterior* probability. A second mass measurement error threshold of ±15 ppm was then applied as part of the post-filtering process after the global data association step for better ion selection.

#### 2.3.2 Peak scoring and filtering

For apex extraction, peaks are characterized according to the following: base peak intensity, *m/z* at the apex, arrival time at the apex, *m/z* width at the base peak, and the arrival time as full width at half maximum. In addition, a peak shape and isotopic distribution score (Ghavidel *et al.* 2014) are calculated to further characterize the quality of the peak and the proximity of the peak to the ideal isotopic distribution of the target molecule. The peak shape score is calculated by quantifying how closely the IMS peak diffusion profile matches the expected Gaussian distribution using the Jaque–Bera Statistical Test (Jarque 1981). Peak shapes deviating significantly from the expected Gaussian distribution are likely to be instrumental or computational artifacts.

The isotopic distribution score is calculated by quantifying the similarity between the theoretical (Jaitly *et al.* 2009) and observed isotopic distribution for each target molecule. The angle between the theoretical and observed isotopic distribution vectors (whose value is normalized to 1) is used as an isotopic matching score (Equation 3). The angle scoring method is preferred to conventional methods (e.g. Euclidean distance or Pearson correlation) for quantifying differences between expected and observed isotopic signatures when differences are large. This attribute is useful for filtering of peaks with a very low probability of being associated with target ions in the preprocessing step.

(3) $$\begin{array}{c} \;\text{cos}\;\theta =\frac{{\vec{I}}_{\tau }\cdot {\vec{I}}_{o}}{\sqrt{\left|{\vec{I}}_{\tau }\right|\cdot \left|{\vec{I}}_{o}\right|}}\\ {\text{Score}}_{\text{isotopic}}=\frac{\theta }{\pi /2} \end{array}$$

In addition, peaks with a summed intensity lower than 3% of the most abundant peak in the extraction window were also discarded as low relative intensity to prevent PIXiE from reporting false conformers. Peaks that did not pass the peak shape threshold, isotopic score threshold or the relative intensity threshold were also discarded in this step.

### 2.4 Global data association

Following the preprocessing steps, the global data association step further removes peaks that are artifacts and determines the link between observed peaks and target molecules. PIXiE makes the following assumptions for this step.

1. Conformers and isobaric species of the target ion(s), are indistinguishable in the *m/z* dimension, and CCSs will be extracted for those ions, if detected.
2. Even under varying electric field, some aspects of the 2D diffusion profiles of a given ion remain the same. For example, the ion cloud distribution is largely determined by the initial pulse into the drift tube and the diffusion that occurs as the ions drift (Siems *et al.* 1994). As a result, the peak profile for a given molecule will broaden predictably (Mason and McDaniel 1988).
3. Peak apices corresponding to the same ion at different electric fields are subject to kinetic theory and can thus be modeled using Equation (1).

To solve the global association problem, we implemented a *K*-shortest path scheme to identify potential association hypotheses constrained by the above assumptions. Next, in the association hypotheses set identified by the *K*-shortest path algorithm, we adapted Bayesian statistics and used maximum *a posterior* estimation (MAP) (Berclaz *et al.* 2011; Siems *et al.* 1994) to test association hypotheses against observed peaks and ultimately select the optimal case.

#### 2.4.1 *K*-shortest path for hypothesis space reduction

If an ion is detected at more than one electric field, an ion path defining the ion’s presence at different electric fields is formed. When large numbers of peaks are observed in the targeted *m/z* space due to low signal to noise ratio or a high number of conformers or co-drifting compounds, the number of possible ion paths that can be formed becomes large enough that the subsequent MAP becomes computationally expensive. To reduce the computational complexity of the MAP calculation, we introduced *K*-shortest path (Berclaz *et al.* 2011) to select the *K* most likely ion paths across the different electric fields. First, we defined an ion transition graph *G* as a weighted and directed graph, where the vertices are the set of all the peaks observed in the targeted *m/z* window: *X* = {*x*_1_, *x*_2_, *x*_3_… *x_i_*… *x_n_*} in all electric fields. Edges are then inserted for every peak in a given electric field to all peaks in the adjacent electric fields (Fig. 1c). Next, the weight of an edge is defined as the negative log of the matching score, *P*_match_(*x_i_*, *x_i_*_+1_), which corresponds to the diffusion profile matching the probability of a pair of peaks in adjacent electric fields (Equation 4). In addition, two non-peak vertices are added to the graph as the source and sink. A hypothetical ion path *T_k_* is thus defined as a path in *G* from the source to the sink (Fig. 1b). Notably, the shorter an ion path, the more consistent are the peaks associated with the path in terms of diffusion profiles as shown by Equation (5). Finding *K* shortest path in the ion transition graph is thus equivalent to finding *T_k_* with the most consistent peak diffusion profiles.

(4) $$\text{weight}(x_{i},x_{i+1})=-\text{log}\;(P_{\text{match}}(x_{i},x_{i+1}))$$

(5) $$\begin{array}{l} |T_{k}|=\sum_{i=1}^{n-1}\text{weight}(x_{i},x_{i+1})\\ \;\;=\sum_{i=1}^{n-1}-\text{log}\;(P_{\text{match}}(x_{i},x_{i+1}))\\ \;\;=-\text{log}\;\left(\prod_{i=1}^{n-1}P_{\mathrm{match}}(x_{i},x_{i+1})\right)\\ \;\;=-\text{log}\;P_{\text{diffusion}}(T_{k}) \end{array}$$

We solve the *K*-shortest paths problem from *G* using the Hoffman–Pavley algorithm (Hoffman and Pavley 1959) to acquire the *K* most likely hypothetical ion paths. Subject to the computing resources available, the number *K* can range from hundreds to thousands if the association result does not converge. *T_k_* would then be combined to form the association hypotheses for the data. The next step is to then use the MAP approach and determine the optimal association of peaks across electric fields.

#### 2.4.2 *A posteriori* probability of association hypothesis

After *K*-shortest path reduces the hypothesis space, the next step is to define and compare the *a posteriori* probability of different association hypotheses. If *X* continues to be the set of all peaks observed in the targeted *m/z* window *X* = {*x*_1_, *x*_2_, *x*_3_…*x_i_*…*x_n_*} in all electric fields, and *T* is the association hypothesis represented by *T* = {*T_k_*}, where *T_k_* ∩ *T_l_* = Ø, then the optimal association hypothesis is one that maximizes its probability given the peaks observed.
(6)
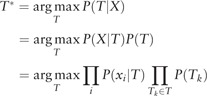

According to Equation (6), the association hypothesis with the highest *a posteriori* probability, *P*(*T_k_*) and *P*(*x_i_* | *T*), needs to be modeled as a function of individual peak profiles, relationships of peaks to ion paths, and the probability of a peak to not reject the association hypothesis. We then model the probability of ion path *P*(*T_k_*) based on Assumptions 2 and 3: where the ion path *T_k_* is more likely to be real if (1) the peak responses share similar diffusion profiles and (2) the arrival time and electric field pairs of the peak comply with the kinetic theory described in Equation (1). *P*(*T_k_*) is thus defined in Supplementary Equation 2 (Supplementary Material S1) as the weighted geometric mean of the correlation of determination value of the least squares fit line (*R*^2^) and the diffusion profile matching the probability for all the observations of an ion (including conformers), which is the *P*_diffusion_ used in the *K* shortest path ion tracking step (Equation 5). For *Pr*(*x_i_* | *T*) we implemented a simplified likelihood model as shown in Supplementary Equation 3 (Supplementary Material S1). If the peak *x_i_* is on one of the paths *T_k_*, we evaluate the arrival time error between the observed peak and what the path *T_k_* predicts. The error corresponds to the degree to which the peak is rejecting hypothetical ion path *T_k_* as a valid ion path. When the peak *x_i_* is not on the ion path *T_k_*, *Pr*(*x_i_* | *T*) is evaluated as δ or the probability of a peak being an artifact or interference within the target *m/z*, which is a constant that can be tuned based on the noise level of the data.

#### 2.4.3 Diffusion profile matching

Both the weight calculation of the ion transition graph and computing *Pr*(*T_k_*) requires computation of the matching probability of base peak diffusion profiles of two peaks measured at different electric fields. We characterize the distance between diffusion profiles of two ions with the following parameters: *Δmzc*, *Δmzw*, *Δmzl*, *Δdtc*, *Δdtw*, *Δdtl*, which correspond to the differences in *m/z* at peak apex, *m/z* full width at half maximum, percentage of peak intensities on the lower *m/z* side of the peak apex, ATD value at peak apex, ATD full width at half maximum and percentage of peak intensities before the peak apex. By calculating the geometric mean of these six parameters weighted according to their impact on the diffusion profile matching, we can obtain a score that favors peaks sharing similar diffusion profiles. This matching score can then be normalized to 1 to model *P*_match_(*x_i_*_,_*x_i_*_+1_) (Supplementary Material S1, Supplementary Equation 1).

### 2.5 CCS calculation and post-filtering

*t*_A_ is calculated for all ion paths in the optimized association hypothesis using the least squares fit of Equation (1) to obtain mobility. Mobility can then be converted to CCS using Equation (2). In addition to the linear simple least squares typically used to calculate ion mobility, PIXiE offers an option to use iteratively reweighted least squares as an alternative with bisquare weight (Wolke and Schwetlick 1988) to account for numerical and instrumental errors in determining the apex of ATD (Supplementary Material S1, Part 2). Although the isotopic filtering and global data association is able to filter out various kinds of artifacts and identify co-drifting compounds, these steps cannot identify the remaining ions as conformers of the target. The identifications might be clear for a simple sample of known composition (e.g. an authentic chemical standard in solution); however, in the case of data from complex samples (e.g. blood plasma), it is still possible that a co-drifting compound could pass the isotopic filter and global association step for CCS extraction. The next step is to use discretional thresholds on multiple scores generated in various steps of the algorithm, such as the isotopic similarity score, mass error in ppm, or the *a posteriori* probability to further identify any remaining co-drifting chemicals. For example, users can establish thresholds such as a mass measurement error ≤10 ppm, or an *a posteriori* probability >0.7. To aid this process, PIXiE generates an analysis database for each batch performed so that users can easily adjust various parameter thresholds for post-filtering through database queries, while viewing the results generated from the optimal association hypothesis.

## 3 Results

### 3.1 An automated tool for DTIMS CCS calculation

We developed PIXiE as an open source, C# software toolkit that automatically extracts arrival times and calculates CCS values for target molecules measured across multiple electric fields in DTIMS-MS experiments. PIXiE implements the preprocessing steps and global data association algorithm described in Figure 1. The primary application for PIXiE is to extract data that can then be independently used in the construction of a reference library containing accurate mass and CCS data for metabolites and other small molecules. To further automate data analysis for an arbitrary amount of targets in a given amount of data, we developed a parallel batch processor function. This allows the user to schedule searches for multiple targets from a set of DTIMS-MS data. The batch processor will run multiple analysis processes, aggregating the results to a single SQLite database consisting of relational tables such as chemical targets, data files, analyses, detected ions and peaks. This database can later be queried directly for quality control, post-processing or visualization purposes. Results after post-processing can then be exported and used to populate an accurate mass and CCS library. Intensive testing of PIXiE on data from DTIMS-MS analyses of 472 small molecules in mixed and standard datasets with seven different IMS electric fields shows that on a windows server with 8-core Intel Xeon X5560 CPU with 2.8 GHz and 25 GB of RAM, PIXiE required 2–5 s to analyze one adduct variant of a target molecule with the maximum *K* in *K*-shortest path set to 3000. The processing time of PIXiE scales linearly with the number of CCS analyses needed.

### 3.2 Peak extraction and filtering

**Fig. 2.**
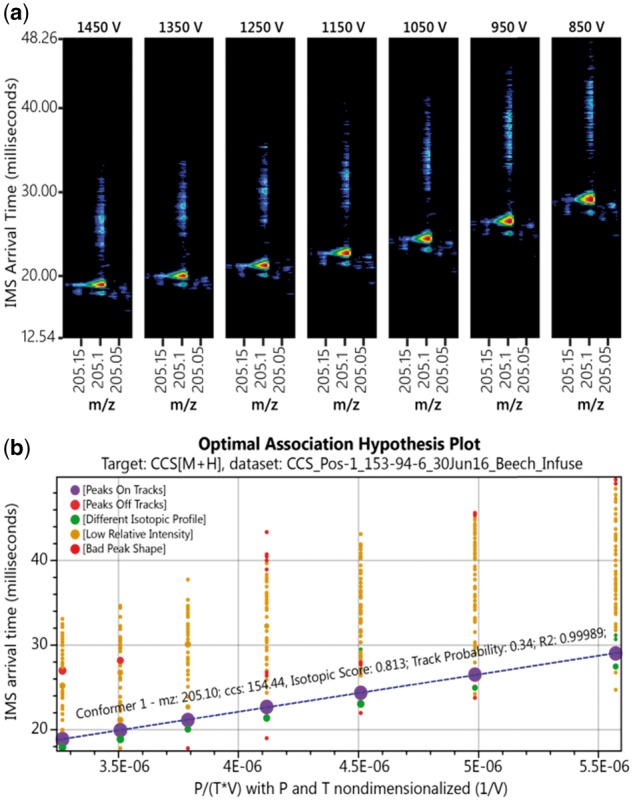
(**a**) Protonated d-tryptophan data at *m/z* 205.0977 Da. As seen in the raw data, as the DTIMS electric field decreases the arrival time of the ions increases. **(b)** Ions were tracked by PIXIE using global data association. The optimal association hypothesis consisted of a single ion path. This association hypothesis had an *a posteriori* probability higher than all other association hypotheses and was thus chosen to explain the observed peaks. Other peaks failed to meet the criteria for identification of the target ion and were thus registered as artifacts. Based on the optimal association hypothesis, a single conformer was reported for the target *m/z* with a CCS value of 154.44 Å^2^

To test the peak extraction and filtering, we manually inspected analyses of 12 standards in positive and negative ionization modes. In addition to listing their optimal association hypotheses in Supplementary Material S2, we here walk through an analysis record from d-tryptophan (PubChem CID 9060) as an example (Table 1). The empirical formula of the target molecule, C_11_H_12_N_2_O_2_, was set in PIXiE to form the corresponding sodiated, protonated and deprotonated adduct ions. Taking the protonated d-tryptophan ion analysis as an example, the accurate mass of the corresponding target *m/z* is 205.0977. PIXiE first grouped the DTIMS-MS data frames into seven electric fields. After averaging the IMS data within the initial *m/z* window of 205.0977 ± 15 ppm, the peak detector found 1, 1, 1, 1, 1, 2 and 2 peaks in electric fields 1 through 7, respectively. Next, the global data association step was applied and the optimal association hypothesis consisted of a single ion. The PIXiE result from peak filtering and peak extraction matches visual inspection of the raw data (Fig. 2). Such peak extraction and filtering information is logged for each optimal association hypothesis to ensure the provenance of PIXiE CCS calculations. In addition, each CCS extracted by PIXiE can be traced to the optimal association hypothesis used and is visualized graphically, as shown in Figure 2b. The optimal association hypothesis plot shows all observed peaks as scatter points according to their arrival time, the value of *p*/(*TV*), and peak intensity.

**Table 1.** Peak statistics averaged across electric fields for the protonated form of d-tryptophan and the filter thresholds used in preprocessing

Criteria	Value	
	Threshold	Measured
	Target detection 1	
	
Intensity score	–	0.9189
Peak shape score	>0.4	0.9037
Isotopic distribution score	>0.4	0.8125
Mass error	<15 ppm	1.8 ppm
*T*_0_	–	4.45 ms
*R*^2^	0.96	0.9999
Mobility (cm^2^/(s*V))	–	1.3930
Cross section (Å^2^)	–	154.4439

### 3.3 Validation of PIXiE extracted CCSs

To verify the accuracy of PIXiE extracted CCSs, we performed a side by side comparison of PIXiE results with those calculated by Agilent’s IM-MS Browser for 12 common metabolites which form 25 total ionic species. The results in Table 2 show that PIXiE-determined CCSs are nearly identical with those calculated by Agilent IM-MS Browser. Slight discrepancies in CCS between the two programs were due to the fact that PIXiE uses the set of points that optimizes the *a posteriori* probability score of an ion path, while Agilent IM-MS Browser uses all available points. This choice in heuristics for PIXiE ensures that specious features are not incorporated in the CCS calculation and preserves confidence in a low false discovery rate during automated batch processing.

**Table 2.** Comparison of PIXiE and Agilent IM-MS browser determined CCSs for 12 metabolites

Metabolite	Adduct	PIXiE CCS (Å^2^)	IM-MS browser CCS (Å^2^)	Difference between programs (%)
(−)-Epinephrine	[M−H]	142.4496	144.0134	1.09
Adenosine	[M+H] (1)	155.555	156.2909	0.47
	[M+H] (2)	168.2187	166.1145	1.27
	[M+Na]	170.7047	170.9785	0.16
	[M−H]	166.5123	168.3901	1.12
Choline	[M+]	117.4588	118.2236	0.65
Cytidine	[M+H]	153.8407	154.2226	0.25
	[M+Na]	162.347	162.6927	0.21
D-Fructose-1,6-BP	[M−H]	156.4098	155.8715	0.35
D-Glucosamine 6-P	[M+H]	154.2323	155.2702	0.67
	[M+Na]	162.9773	161.5521	0.88
	[M−H]	150.5087	150.481	0.02
D-Tryptophan	[M+H]	154.4439	153.8472	0.39
	[M+Na]	149.2501	150.1552	0.60
	[M−H]	155.872	156.5406	0.43
Folic acid	[M+H]	195.2117	195.2644	0.03
	[M+Na]	203.1976	204.6301	0.70
	[M−H]	194.6636	194.4258	0.12
NAD	[M+H]	227.936	226.236	0.75
	[M+Na]	223.3434	223.4154	0.03
	[M−H]	227.1015	227.8383	0.32
Sucrose	[M+Na]	174.6098	174.2231	0.22
	[M−H]	170.1421	169.641	0.30
Taurine	[M+H]	139.7391	140.4202	0.49
UDP-Galactose	[M−H]	210.8353	211.6699	0.39

### 3.4 Algorithm robustness

To evaluate how robust PIXiE was for determining optimal association hypotheses, we tested the algorithm on datasets containing multiple conformers. We also observed cases where the small molecules would form non-covalent dimers and break into monomers prior to detection, so these were also evaluated. For these molecules, multiple peaks were observed several times for a target (Supplementary Material S2). However, in the analyses of authentic reference materials we have yet to encounter a case where the sample complexity at a single mass range was too complex for PIXiE to determine a reasonable optimal association hypothesis. There are times though that PIXiE ignores certain high-leverage peaks from the optimal association hypothesis as these peaks can sometimes reduce the *a posterior* probability of the association hypothesis. This behavior can be seen in Supplementary Material S2 for NAD [M+H], taurine [M+H], adenosine [M+Na] and others.

### 3.5 Limitations

Although PIXiE can reliably locate ions in the data that match the mass and isotopic pattern specified by the target, it does not offer absolute identification nor can PIXiE draw a line between conformers and dimers. For the use of library construction purposes, sample purity should be high to ensure corresponding high confidence in the determined CCS value. Extraction of data for library building is best conducted using conservative parameters (e.g. low mass error threshold, high isotopic score threshold, etc.) to limit the probability of incorrect association of peaks with target molecules.

## 4 Conclusion

The use of CCS as a metric for confident identification of metabolites and other small molecules in metabolomics studies has high potential. However, the difficulty of implementing automated data analysis has limited the throughput of multi-electric field DTIMS-MS CCS extraction. To address these challenges, we have developed PIXiE for reliably extracting CCSs from multi-electric field DTIMS-MS data. Using the global data association algorithm, PIXiE was able to track targets in the presence of co-drifting ions representing conformers or chemical noise. The optimal association hypothesis distills ions from the collected DTIMS-MS data having *m/z* consistent with the target molecule, isotopic profiles similar to the expected isotopic profile of the target, and peak responses across electric fields that comply with ion kinetic theory (Mason and McDaniel 1988). Most importantly, CCS values can easily be calculated for each ion distilled based on the arrival times at the different electric fields. Arrival times can then be documented using the measured arrival time and subtracting the time an ion spends outside the drift tube, which is independently evaluated in the least squares step. For applications where multi-electric field DTIMS-MS measurements are performed, PIXiE increases the throughput of CCS determination.

## Funding

This research was supported by the Laboratory Directed Research and Development (LDRD) program at the Pacific Northwest National Laboratory (PNNL) and is a contribution of the Global Forensic Chemical Exposure Assessment for the Environmental Exposome project and the Microbiomes in Transition Initiative. The work was also supported by the National Institute of Environmental Health Sciences of the National Institutes of Health [R01 ES022190]. Portions of this research utilized capabilities developed with support from the Department of Energy Office of Biological and Environmental Research Genomic Science Program under the PNNL Pan-omics Program. DTIMS-MS analyses were performed in the Environmental Molecular Sciences Laboratory, a national scientific user facility sponsored by the U.S. Department of Energy (DOE) Office of Biological and Environmental Research and located at PNNL. PNNL is a multi-program national laboratory operated by Battelle for the DOE under Contract DE-AC05-76RLO 1830.

*Conflict of Interest*: none declared.

## Supplementary Material

Supplementary Data

Supplementary Data
