# Supplementary material for: PIXiE: an algorithm for automated ion mobility arrival time extraction and collision cross section calculation using global data association
Source: Bioinformatics. 2017 May 15;33(17):2715–22. doi: 10.1093/bioinformatics/btx305 (PMC5860068; doi:10.1093/bioinformatics/btx305)
Supplement: Supplementary Data [file btx305_supplementary_pixie-s1-methods_bioinformatics_revised.docx]

### PIXiE: An Algorithm for Automated Ion Mobility Arrival Time Extraction and Collision Cross Section Calculation using Global Data Association Supplemental 1

Jian Ma^1^, Cameron P. Casey^1^, Xueyun Zheng^1^, Yehia M. Ibrahim^1^, Christopher S. Wilkins^1^, Ryan S. Renslow^1^, Dennis G. Thomas^1^, Samuel H. Payne^1^, Matthew E. Monroe^1^, Richard D. Smith^1^, Justin G. Teeguarden^1,2^, Erin S. Baker^1,*^ and Thomas O. Metz^1,*^

^1^Biological Sciences Division, Pacific Northwest National Laboratory, Richland, WA 99352, ^2^Department of Environmental and Molecular Toxicology, Oregon State University, Corvallis, OR 93771

*To whom correspondence should be addressed.

### 1. *Supplemental* *Equations*

Diffusion profile matching probability modeling. The probability of diffusion profiles matching P_match_ , defined in section 2.4.1, is modelled as Supplementary Eqn.1 in PIXiE. A diffusion profile matching score is calculated as the weighted geometric mean of intensity matching score, diffusion profile matching score and *m/z* matching score. P_match_ is the diffusion profile matching score normalized to 1.

|  | $\begin{matrix} Score_{match}(u,v)=e^{w_{\mathrm{int}ensity}\cdot\ln(Score_{\mathrm{int}ensity}(u,v))+w_{diffusion}\cdot\ln(Score_{diffusion}(u,v))+w_{mz}\cdot\ln(Score_{mz}(u,v))} \\ P_{match}(u,v)=1-e^{-a\cdot score_{match}(u,v)} \end{matrix}$ | (1) |
| --- | --- | --- |

score_intensity_(u,v), score_diffusion_(u,v) and score_mz_(u,v) are a set a scores used to model how close 2 adjacent diffusion profiles compare in intensity, shape of the diffusion profiles and *m/z* values. The exact definitions of those scores and their weights can be implemented in various ways but PIXiE chose a very simple set of definitions that is able to penalize large deviations in either intensity, m/z or peak shape. We did not further analyze the effects of varying the scoring methods definitions beyond the following, after getting favorable test results form our test data set.

$\mathrm{Score}_{intensity}\left( u,v \right)=1-(|I_{u} - I_{v}|)/max(I_{u}, I_{v})$, whereas I_u_, I_v_ are the intensity of u, v peaks respectively.

$\mathrm{Score}_{diffusion}\left( u,v \right)=1- e^{(-b*{\Delta width}_{mz})}$, where Δwidth*_m/z_* is the difference between peak widths in *m/z* dimensions. b is an arbitrarily defined scaling factor. Note that we reduced peak shape matching to comparing *m/z* widths between 2 peaks here for ease of implementation.

$\mathrm{Score}_{mz}\left( u,v \right)=1- e^{(-c*\Delta mz)}$, where Δ*m/*z is the difference in *m/z* between the peak apex, and c is an arbitrarily defined scaling factor.

MAP Bayesian probability models used in PIXiE. The probability of an ion path P(T_k_) is modelled as Eqn.2 in PIXiE. The ion path score is calculated as weighted geometric mean of the overall diffusion profile matching probability P_diffusion_(T_k_), and a function of the R-squared value of the ion path T_k_.

|  | 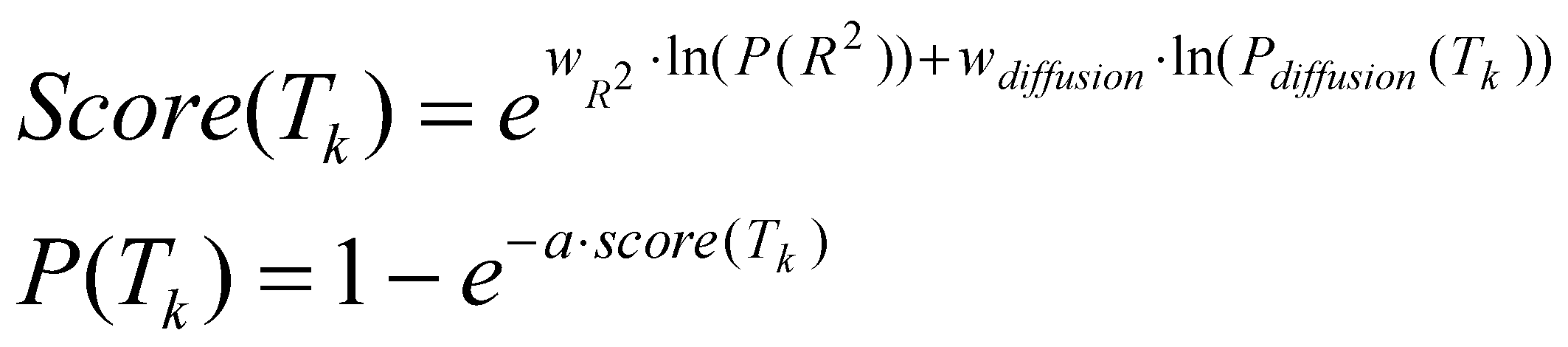 | (2) |
| --- | --- | --- |

The likelihood of an observed peak x_i_ given an association hypothesis T is modeled with Eqn.3, if x_i_ does not land on any ion paths in the association hypothesis, the likelihood is a constant δ corresponding to the overall noise level of the dataset. If x_i_ does land on an ion path T_k_ within T, the likelihood P(x_i_ | T) becomes a function of the residue of the observed peak.

|  | 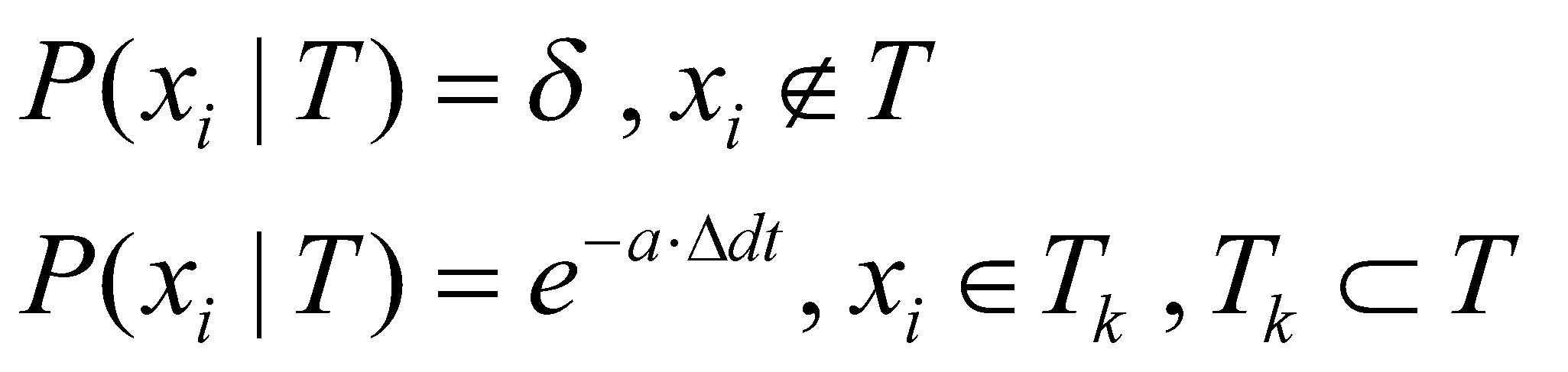 | (3) |
| --- | --- | --- |

### *2. The iteratively reweighted least squares option*

PIXiE also gives an option to deviate from the traditional multi-electric field collision cross section extraction method to account for certain error models. Using the simple linear least squares, the uncertainty of drift time measurement in high-leverage peaks could contribute an unproportioned amount of measurement uncertainty in collision cross section, e.g., one TOF pulse of drift time error of about 160μs at the highest electric field could contribute ~10 Å^2^ of error in collision cross section. To mitigate this effect PIXiE introduced an option to replace traditional simple least squares with iteratively reweighted least squares (IRLS) (Wolke and Schwetlick, 1988). This option has two benefits:

1. IRLS redistributes each electric field’s contribution to the final CCS result evenly. In theory it should improve both accuracy and precision, the scale of which depends on types of error present.
2. IRLS especially acts as a countermeasure to numerical and measurement errors in drift time/temperature/pressure measurements in high leverage voltage groups.

To evaluate the impact of iteratively reweighted least squares on the calculation of collision cross sections, we compared the collision cross section values that PIXiE generates to in silico prediction values. The average percentage error between predicted and experimental values was 5.2%. Turning on iteratively reweighted least squares reduces the average percentage error to 4.5%. A detailed correlation is plotted in **Fig. S1**. As a result, we conclude that turning on the IRLS option reduces measurement uncertainties. The IRLS option is especially recommended for experiments conducted with uneven electric field voltages.


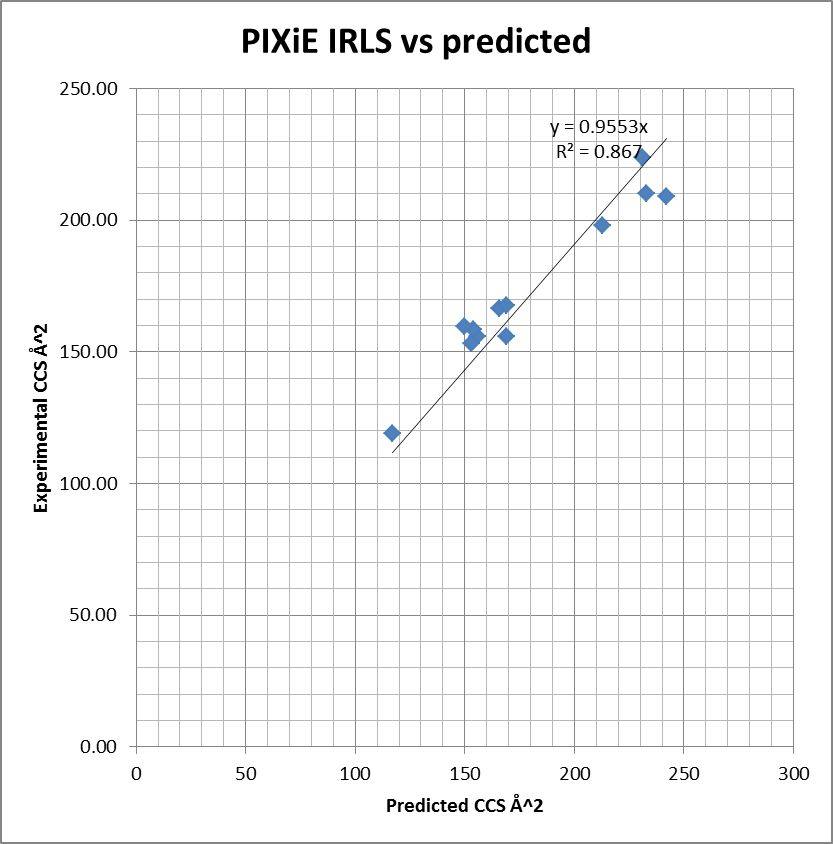

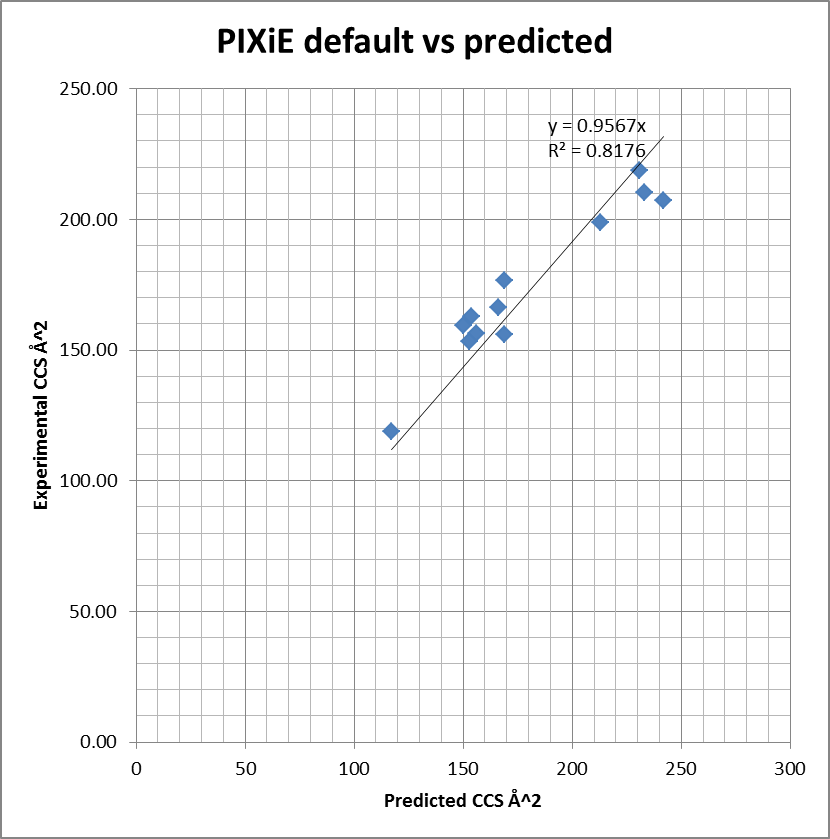


|  |  |
| --- | --- |
| **Figure S1. (a) Correlation plot of PIXiE CCS vs NWChem predicted CCS with the IRLS option off. (b) Correlation plot of PIXiE CCS vs NWChem predicted CCS with the IRLS option on.** | |

***3. Materials, Sample Preparation and DTIMS-MS Analysis:***

Authentic standards were purchased from Sigma Aldrich (St. Louis, MO) and prepared in 100% methanol to a concentration of 1 µM. The standards were each analyzed using an Agilent 6560 ion mobility-quadrupole time of flight mass spectrometry (IM-QTOF MS) platform (May *et al.*, 2014; Ibrahim *et al.*, 2015). Briefly, after electrospray ionization, the ions were passed through the inlet glass capillary, focused by a high pressure ion funnel, and accumulated in a lower pressure ion funnel trap (IFT). Ions were then pulsed into the 90 cm-long IMS drift tube filled with ~ 4 torr of nitrogen gas, where they travel under the influence of seven weak electric fields (10-20 V/cm). Ions exiting the drift tube were refocused by a rear ion funnel prior to QTOF MS detection and their arrival times were recorded and used for cross section calculation.

**References**

Ibrahim, Y. M., et al (2015). Development of a New Ion Mobility (Quadrupole) Time-of-Flight Mass Spectrometer. *Int J Mass Spectrom*, *377*, 655-662.

May, J. C., et al. (2014). Conformational Ordering of Biomolecules in the Gas Phase: Nitrogen Collision Cross Sections Measured on a Prototype High Resolution Drift Tube Ion Mobility-Mass Spectrometer. *Anal. Chem.*, *86*, 2107-2116.

Wolke, R. and H. Schwetlick (1988). Iteratively Reweighted Least Squares: Algorithms, Convergence Analysis, and Numerical Comparisons. *SIAM Journal on Scientific and Statistical Computing*,. **9**(5): 907-921.
