# Supplementary material for: PIXiE: an algorithm for automated ion mobility arrival time extraction and collision cross section calculation using global data association
Source: Bioinformatics. 2017 May 15;33(17):2715–22. doi: 10.1093/bioinformatics/btx305 (PMC5860068; doi:10.1093/bioinformatics/btx305)
Supplement: Supplementary Data [file btx305_supplementary_pixie-s2-association_bioinformatics_revised.docx]

### PIXiE: An Algorithm for Automated Ion Mobility Arrival Time Extraction and Collision Cross Section Calculation using Global Data Association Supplemental 2

Jian Ma^1^, Cameron P. Casey^1^, Xueyun Zheng^1^, Yehia M. Ibrahim^1^, Christopher S. Wilkins^1^, Ryan S. Renslow^1^, Dennis G. Thomas^1^, Samuel H. Payne^1^, Matthew E. Monroe^1^, Richard D. Smith^1^, Justin G. Teeguarden^1,2^, Erin S. Baker^1,*^ and Thomas O. Metz^1,*^

^1^Biological Sciences Division, Pacific Northwest National Laboratory, Richland, WA 99352, ^2^Department of Environmental and Molecular Toxicology, Oregon State University, Corvallis, OR 93771

*To whom correspondence should be addressed.

**Examples of global data association results from DT-IMS-MS analyses of endogenous metabolites and exogenous chemicals.** As the drift tube voltage decreases, arrival time of the ions increases. The following plots show the optimal association hypotheses of each analysis. Each connected purple dot represents a hypothetical ion path. Peaks that were filtered in the preprocessing step were marked with green, yellow, and maroon.

### The first example shows three conformers of a dimer of glutathione with the loss of a hydrogen atom in negative ionization mode. The extracted features and association hypotheses selected (bottom) may be compared with the heat map of the raw data (top).

### Glutathione dimer [2M-H]

**
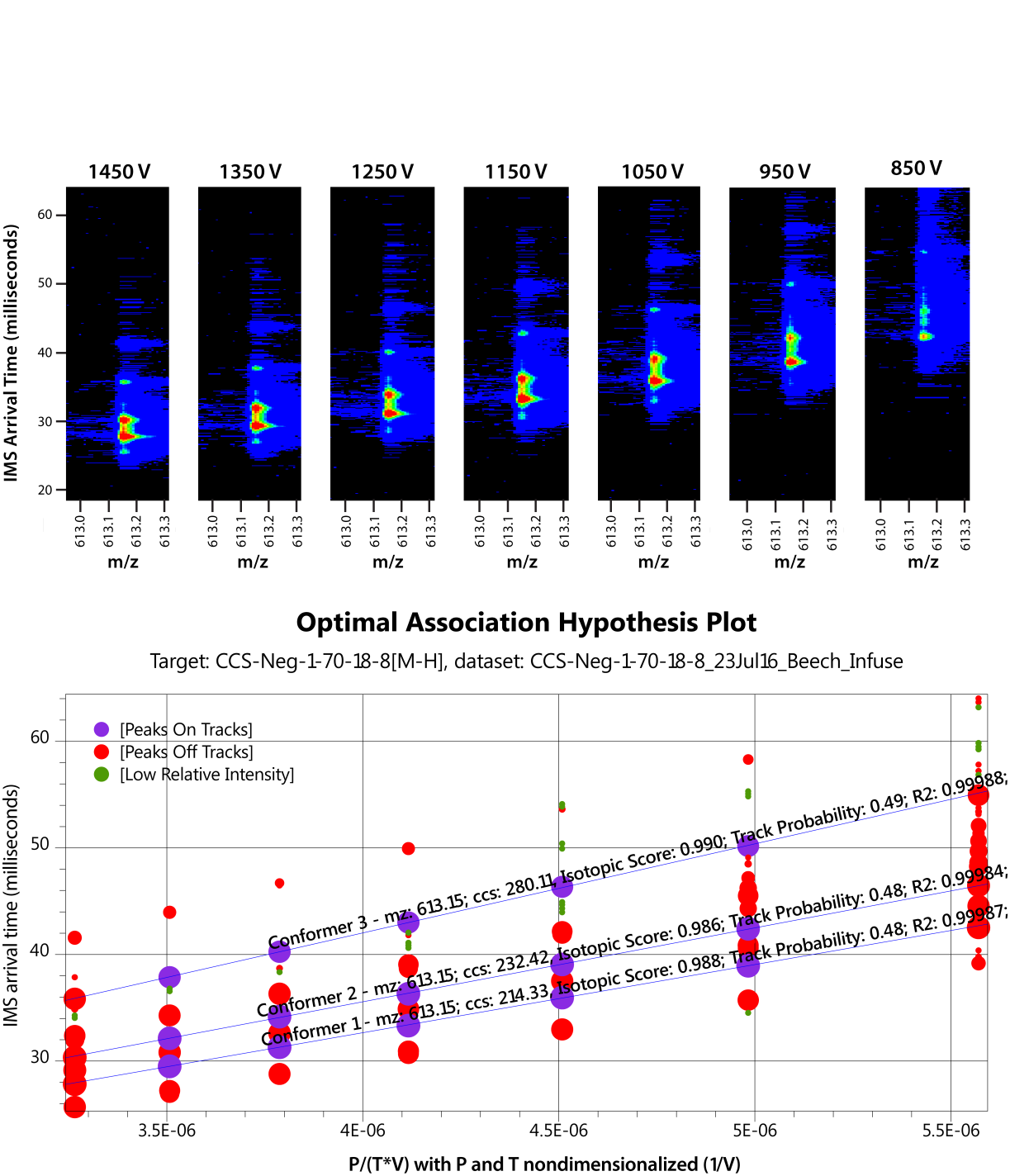
**

**Choline [M+]**

**
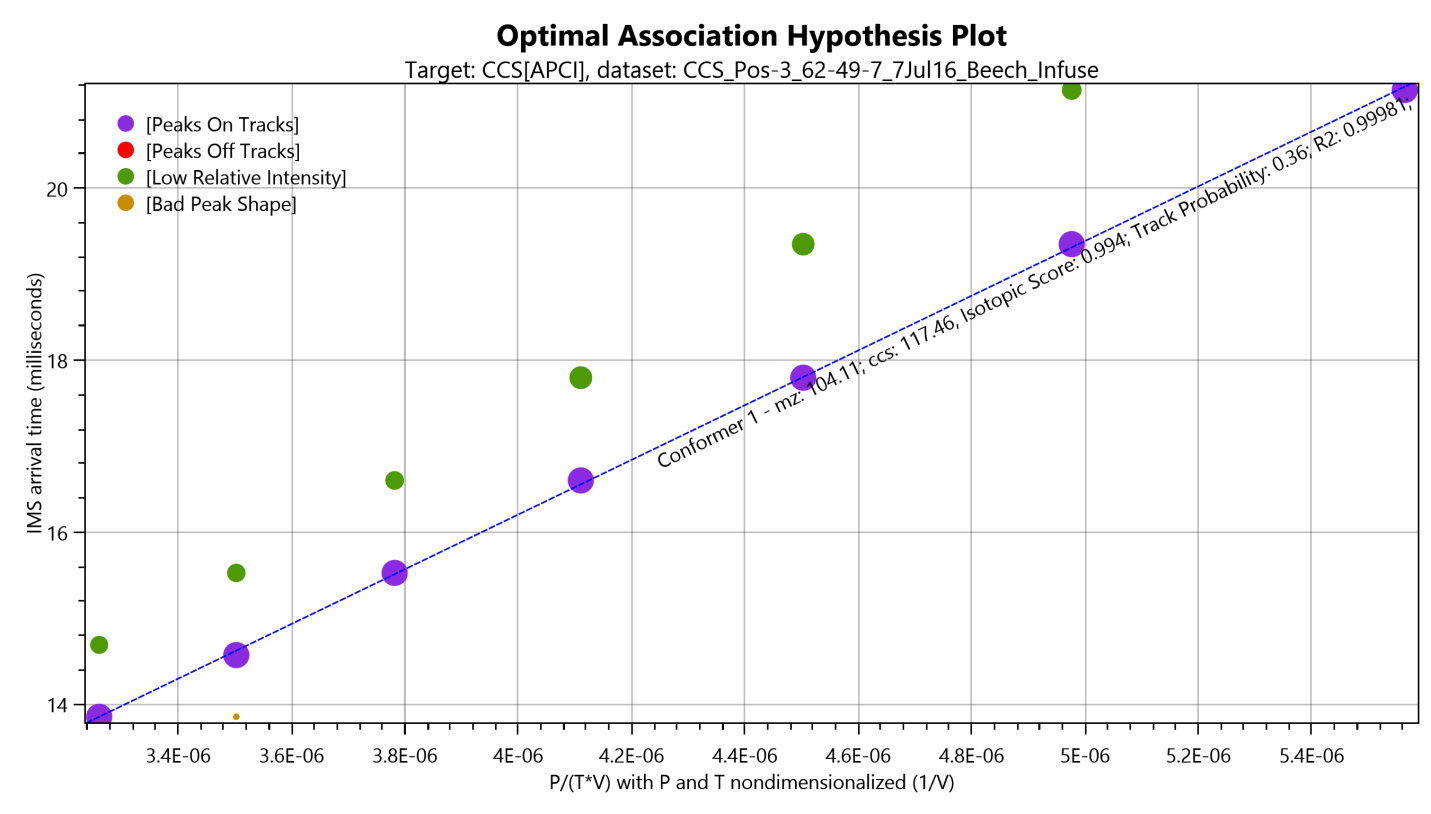
NAD [M+H]
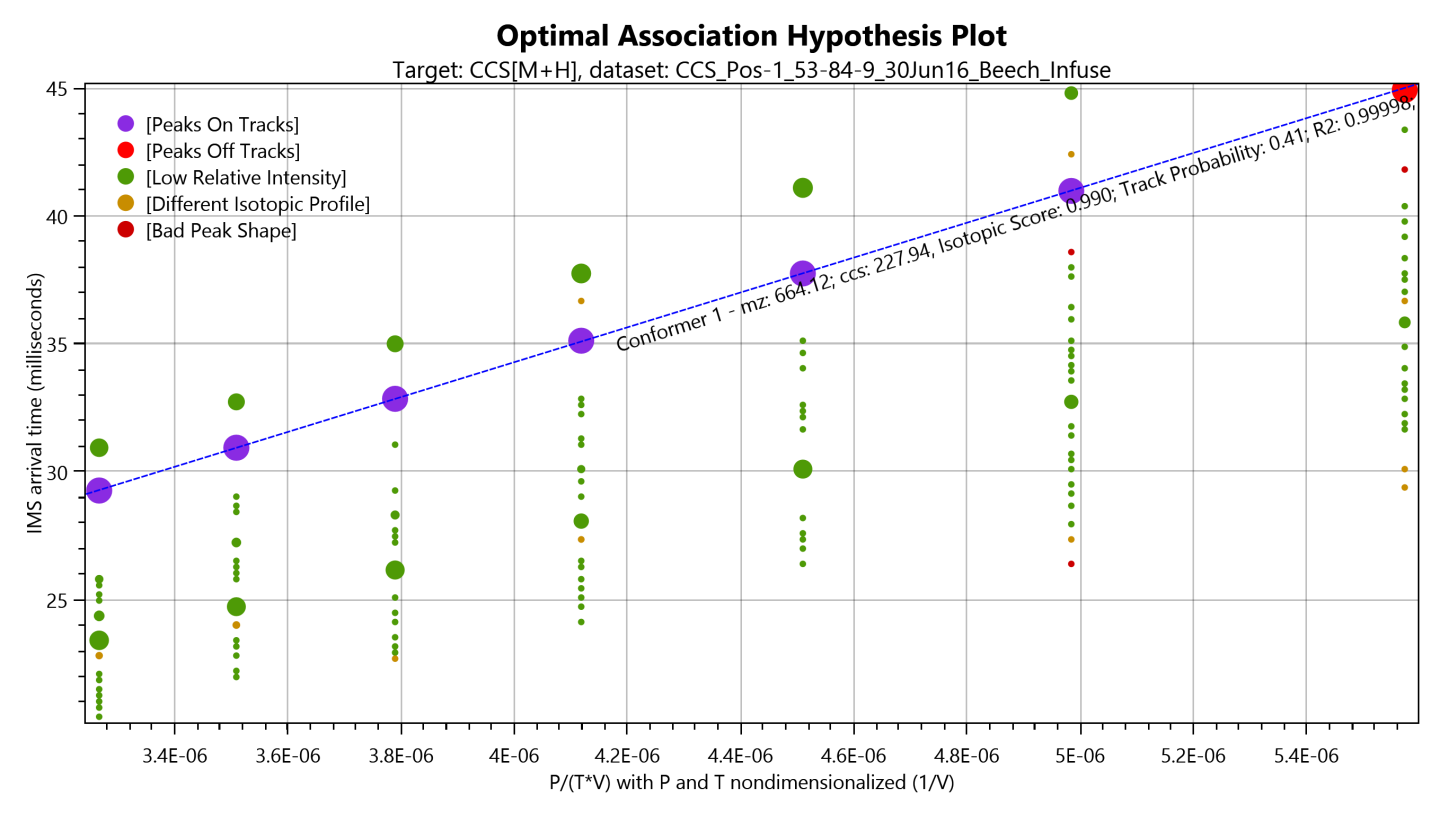
**

**Cytidine [M+H]
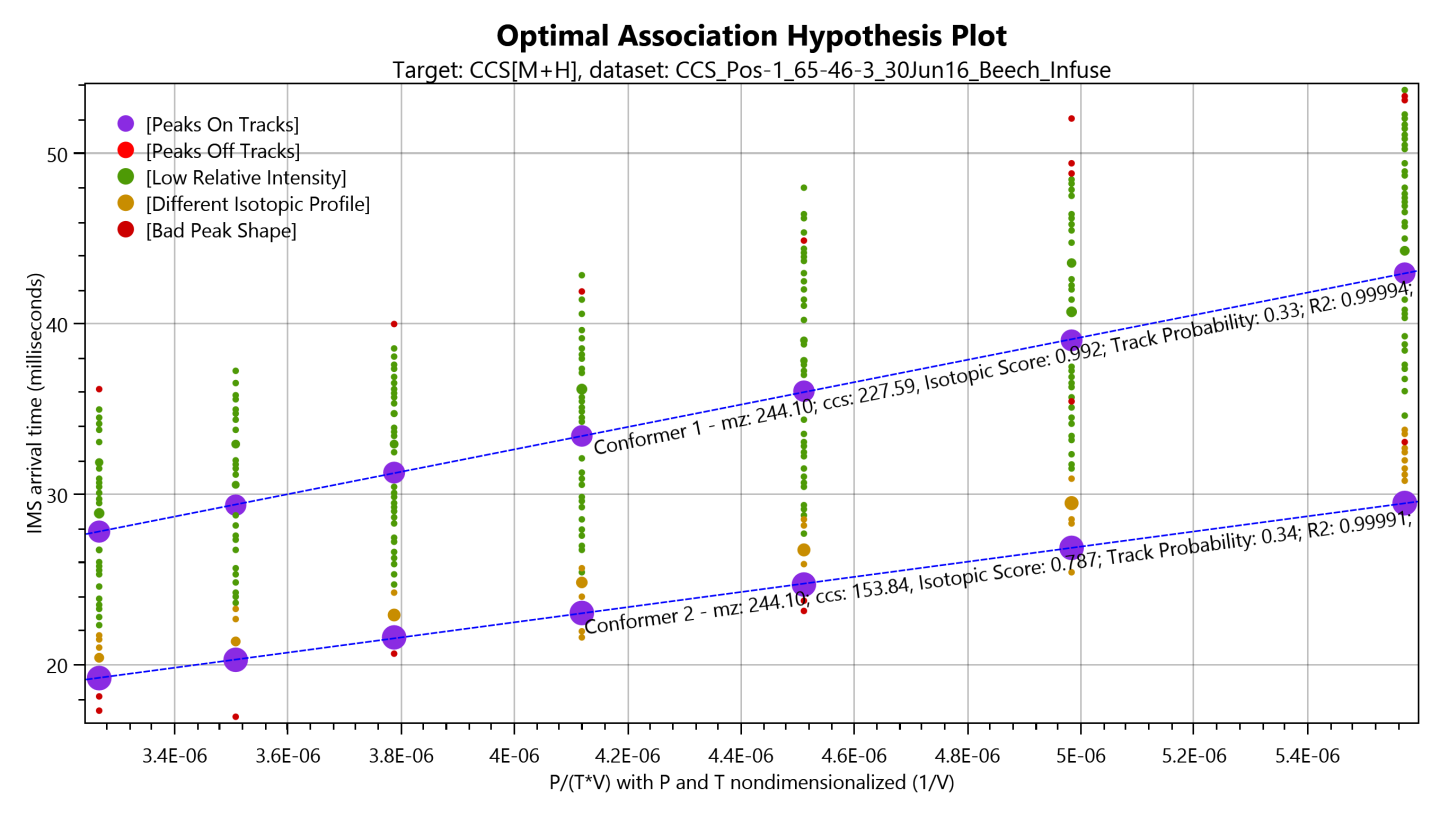
Taurine [M+H]
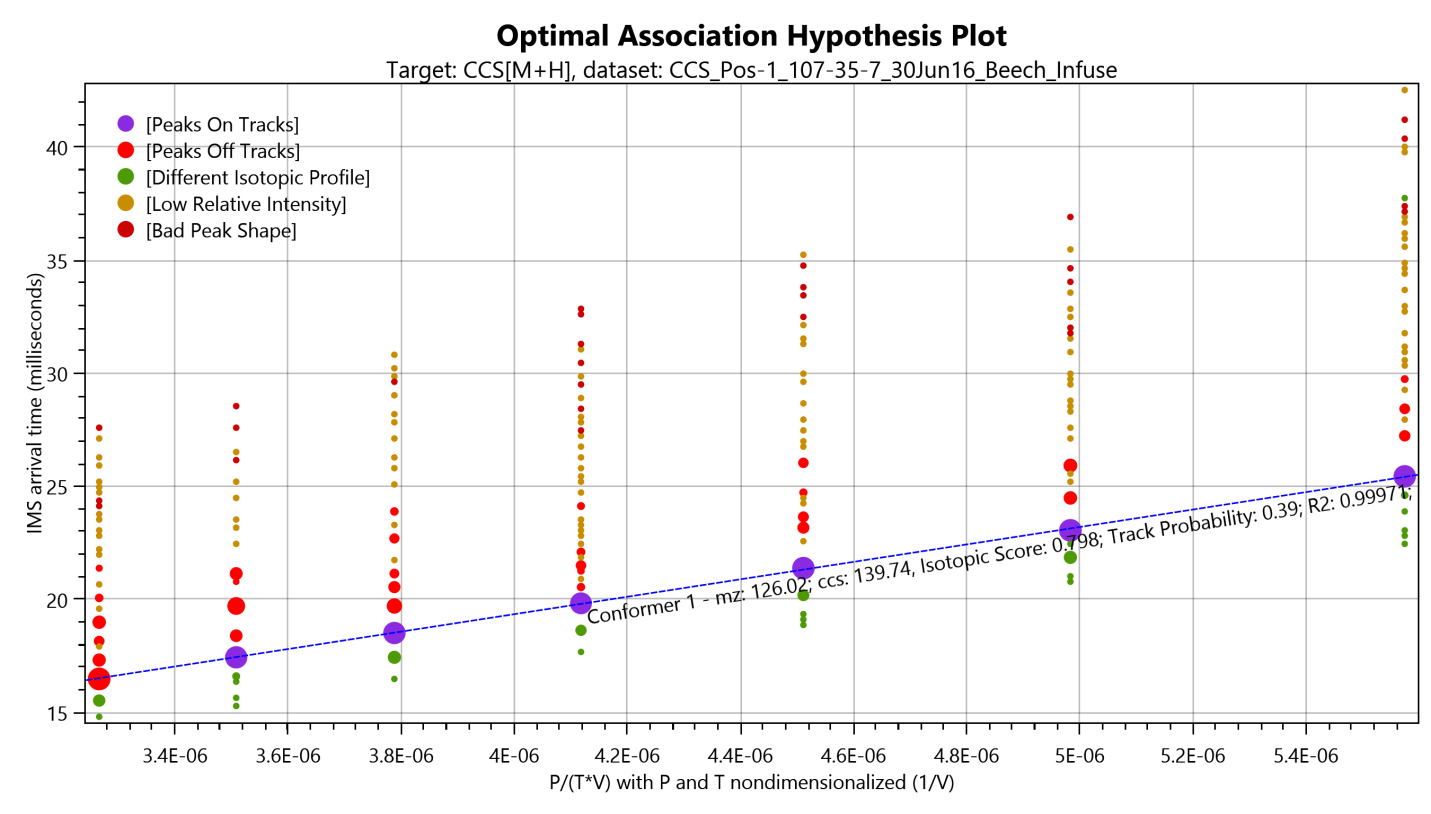
**

**D-Tryptophan [M+H]
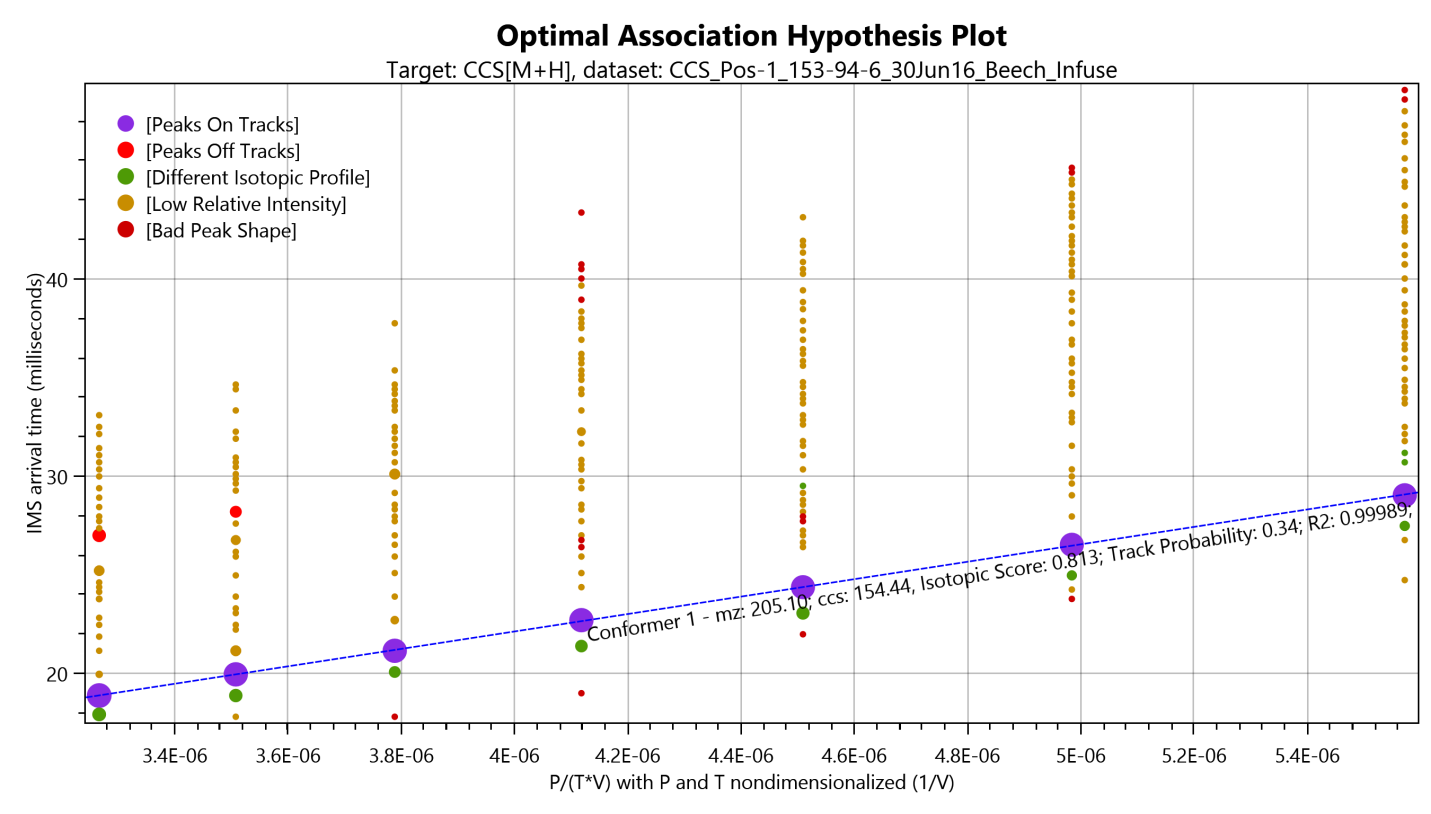
Folic acid [M+H]
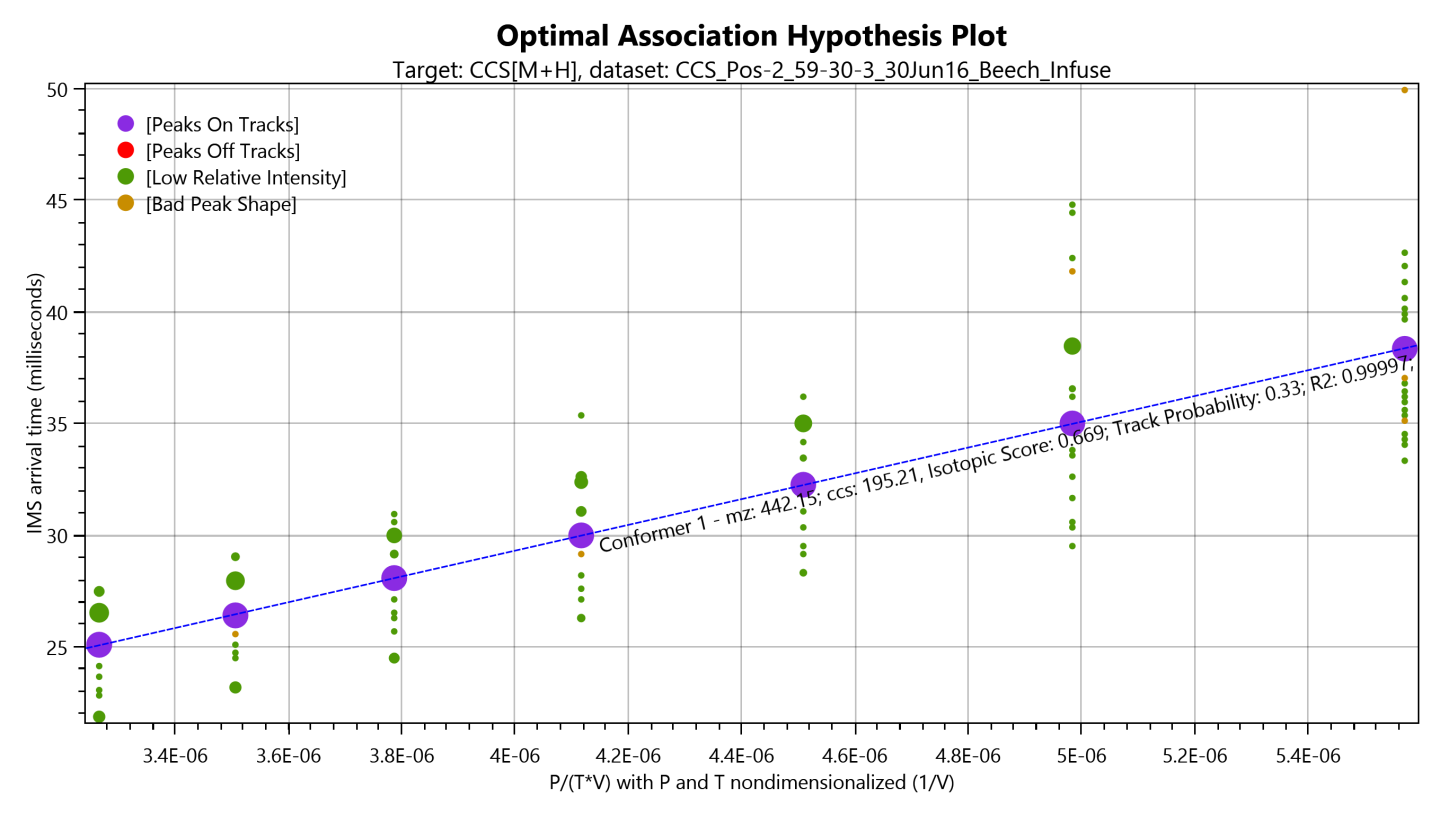
**

**Adenosine [M+H]
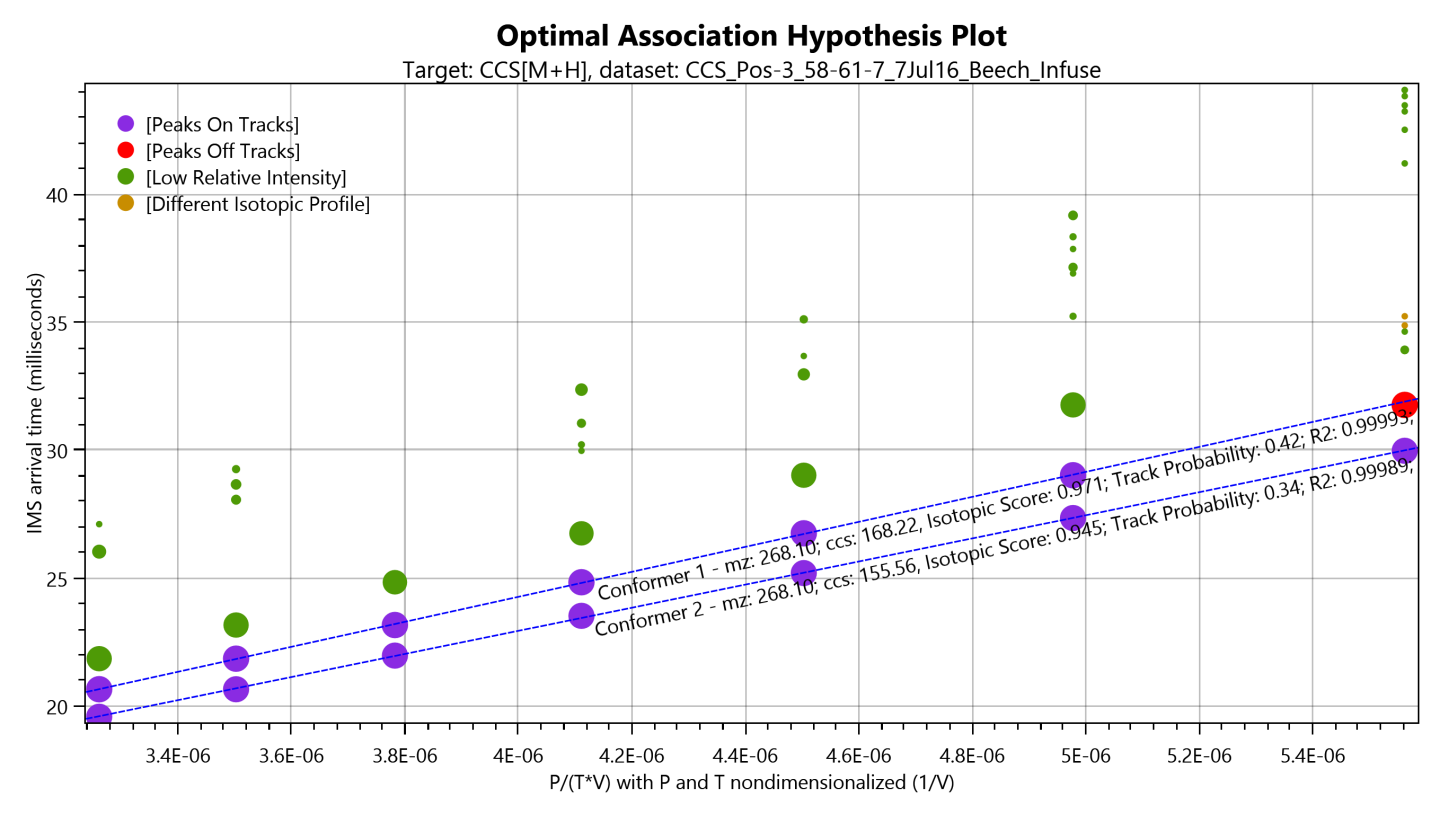
D-Glucosamine 6-phosphate
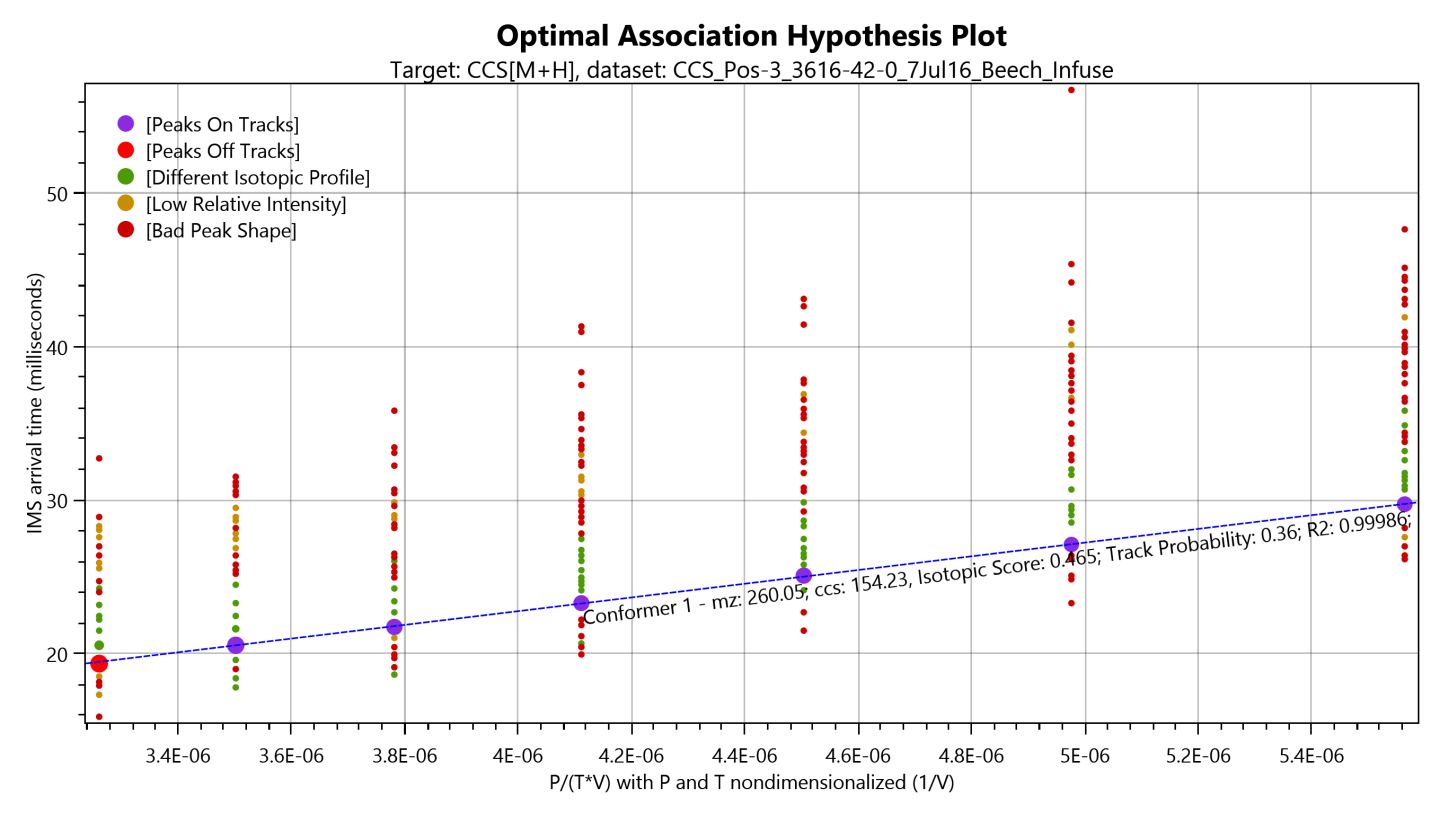
**

**NAD [M+Na]
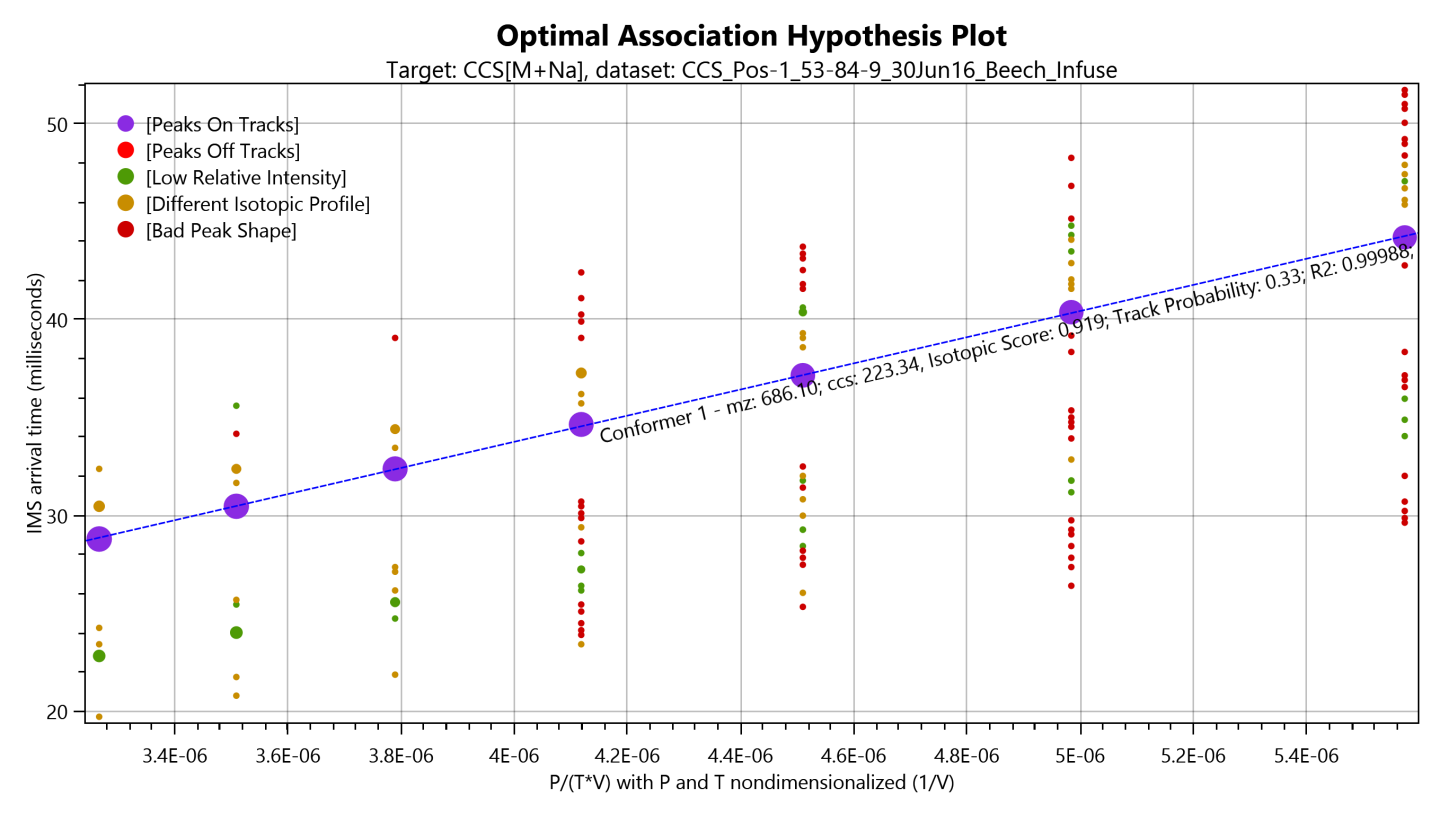
Sucrose [M+Na]
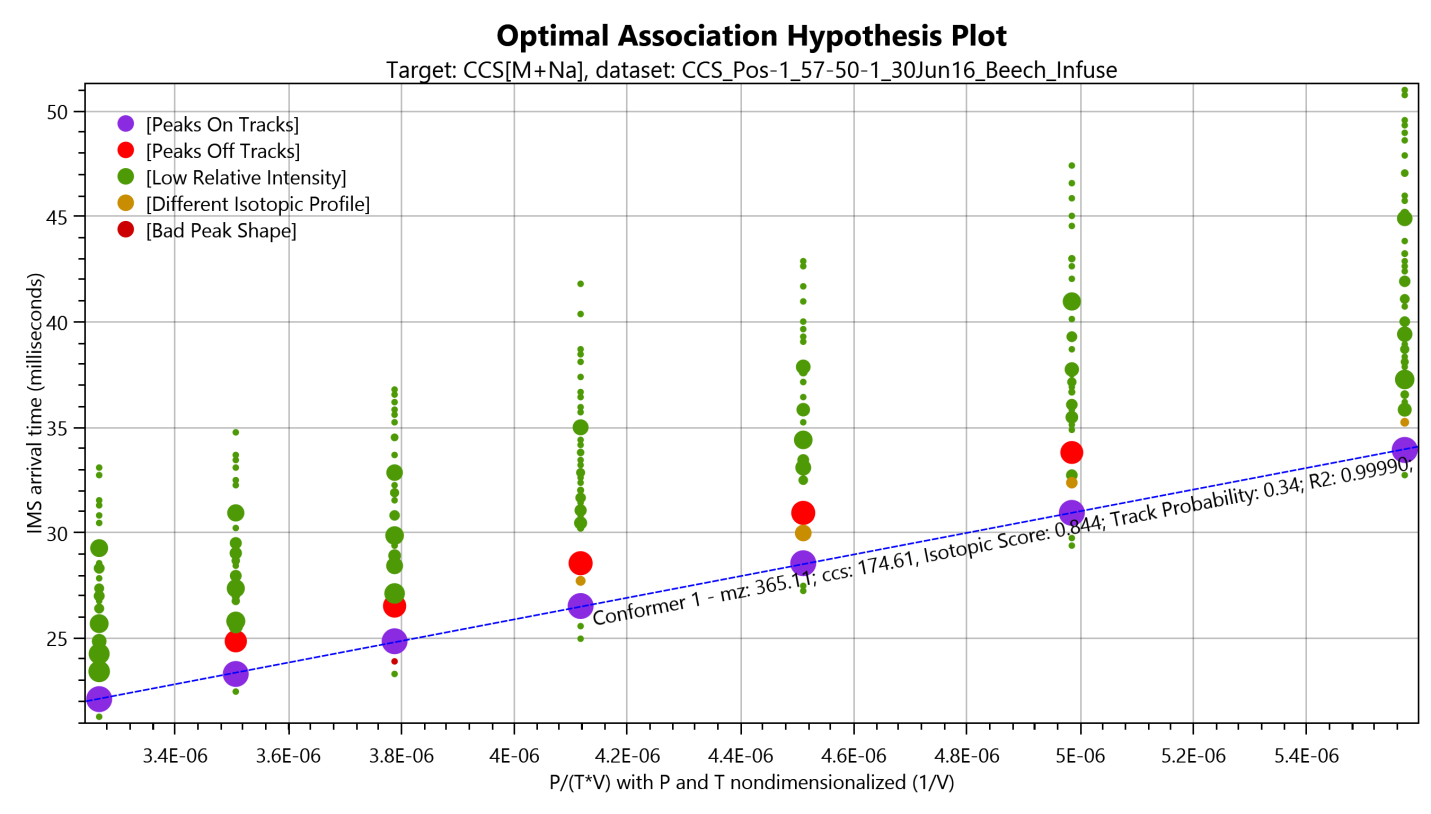
**

**Cytidine [M+Na]
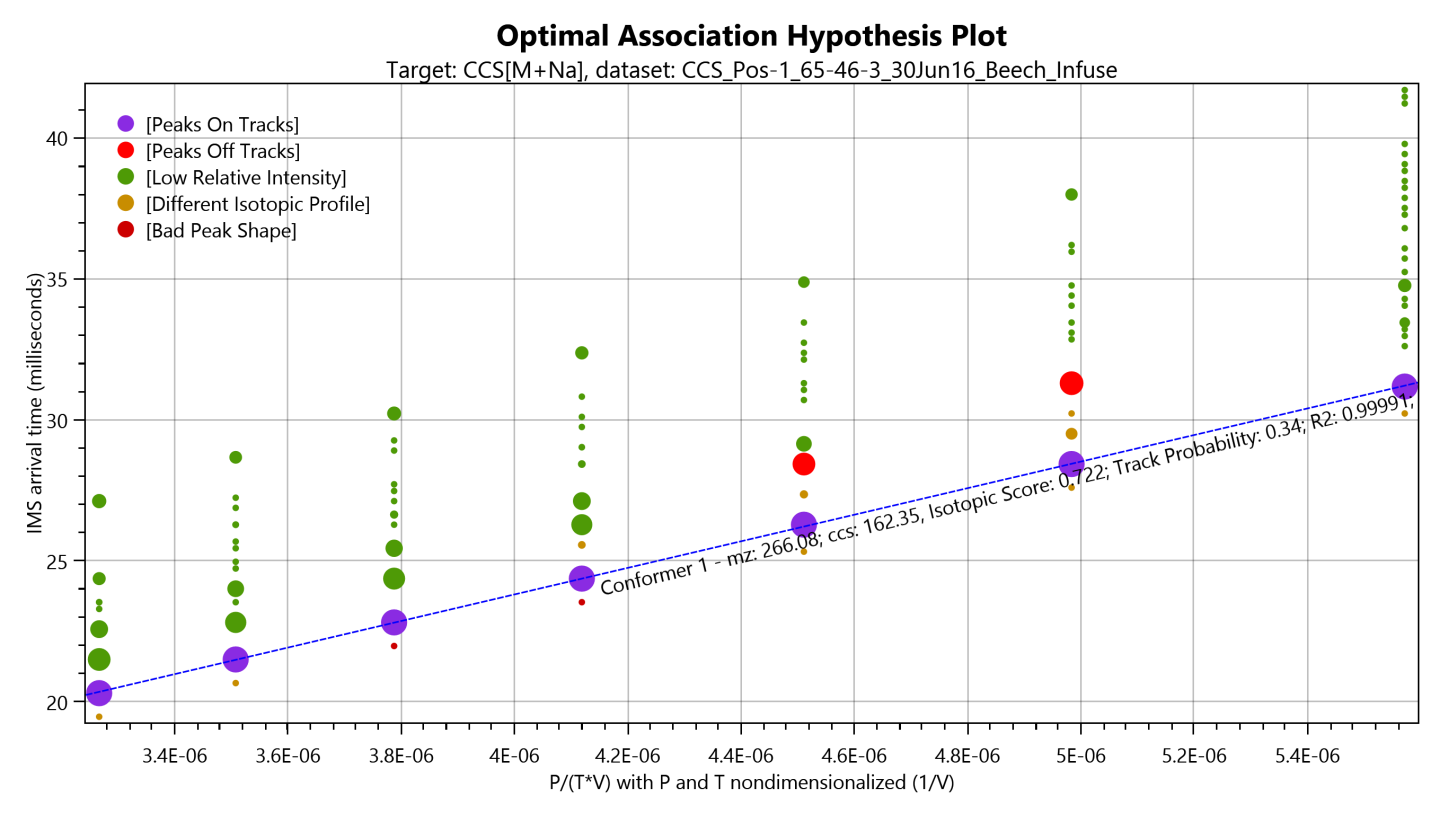
D-Tryptophan [M+Na]
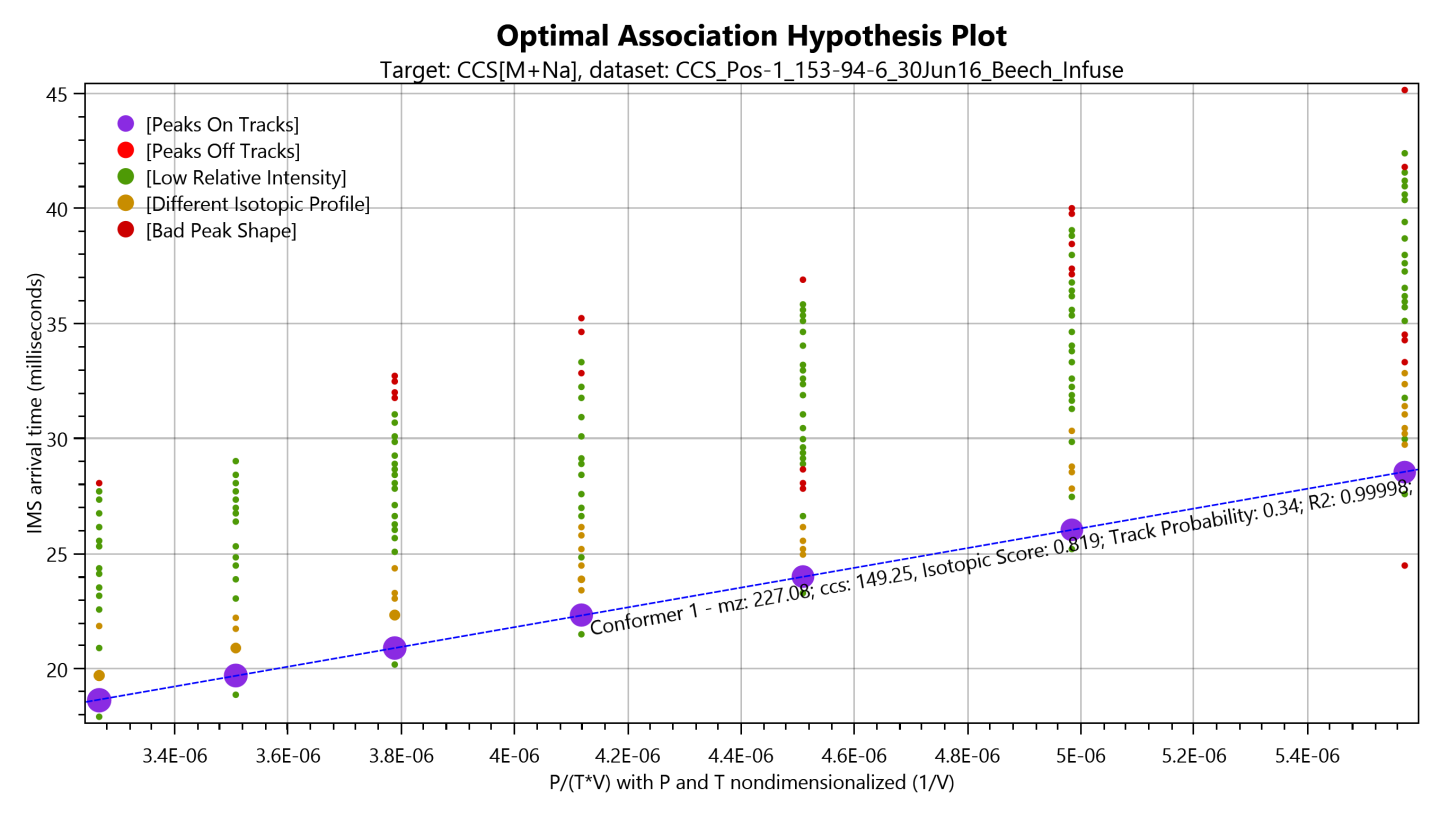
**

**Folic acid [M+Na]
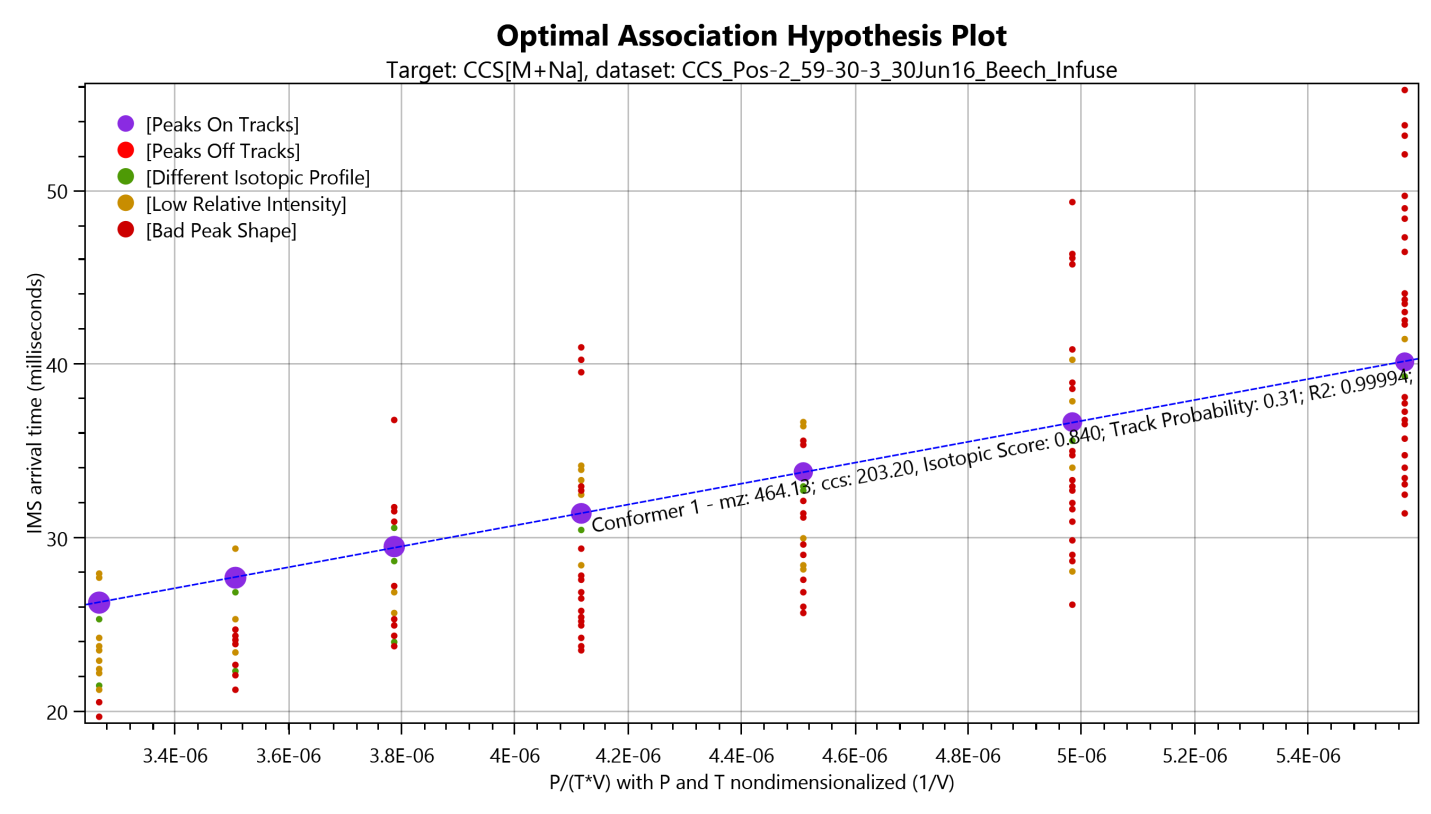
Adenosine [M+Na}
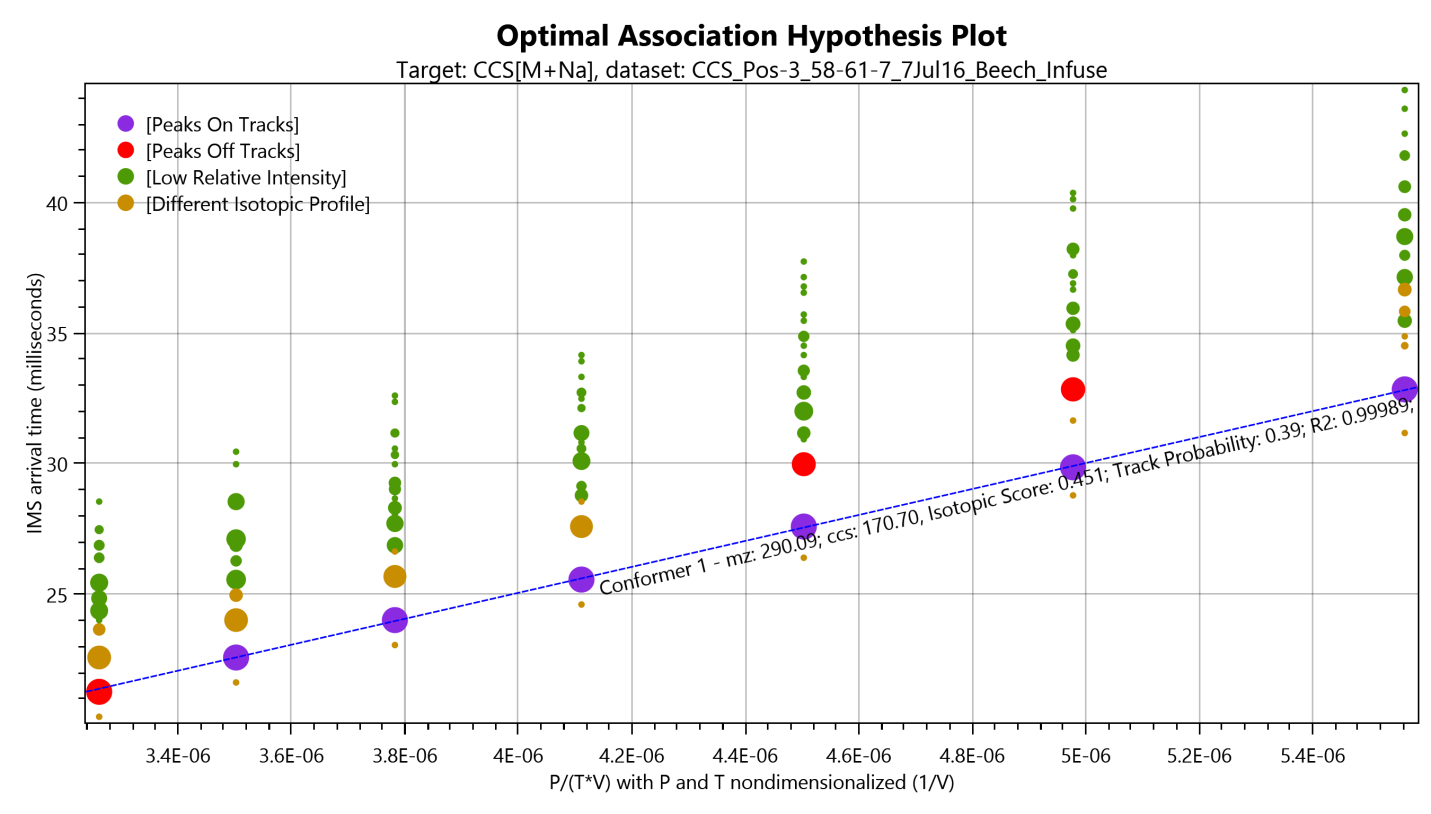
**

**D-Glucosamine 6-phosphate [M+Na]
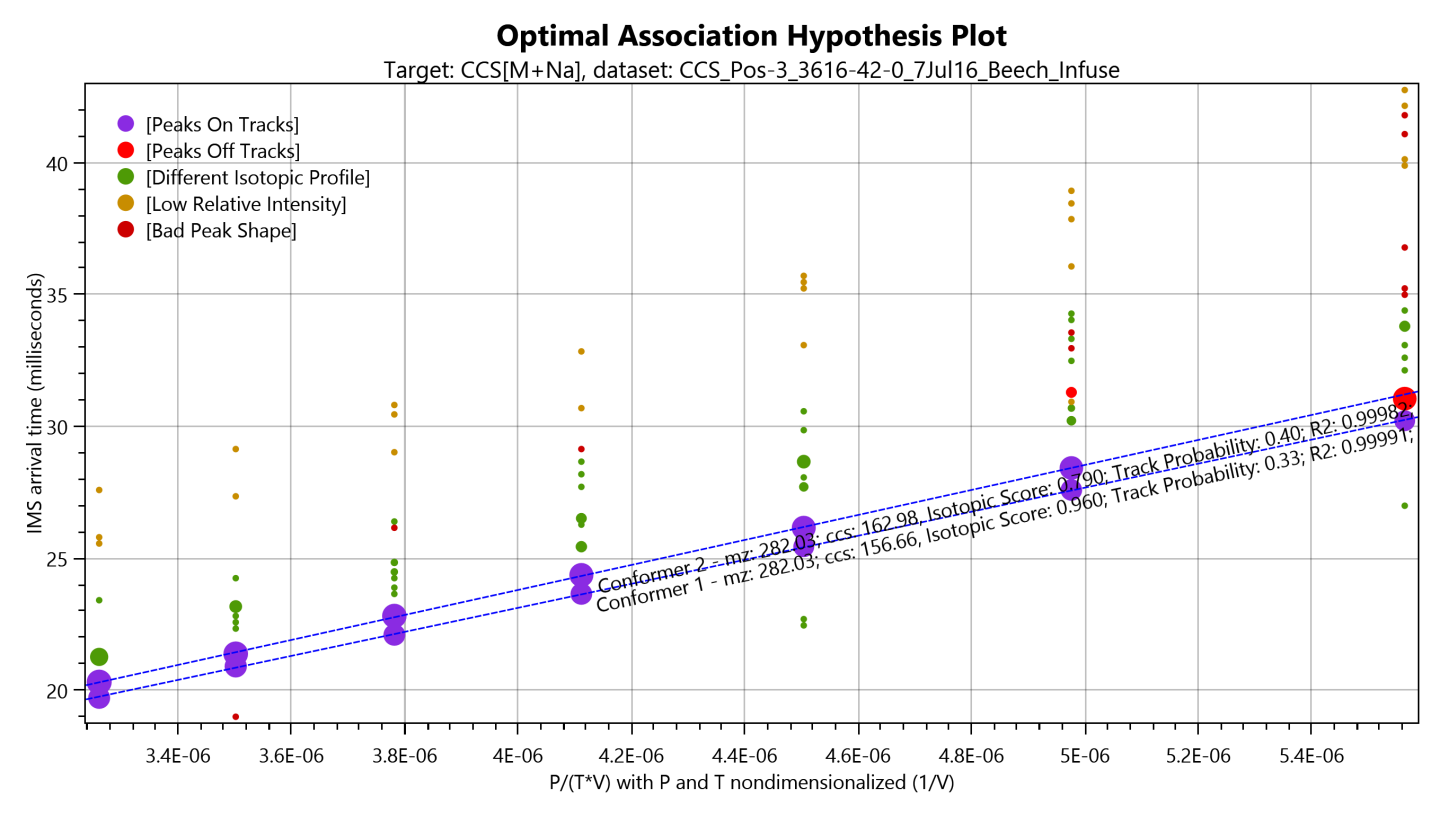
Folic acid [M-H]
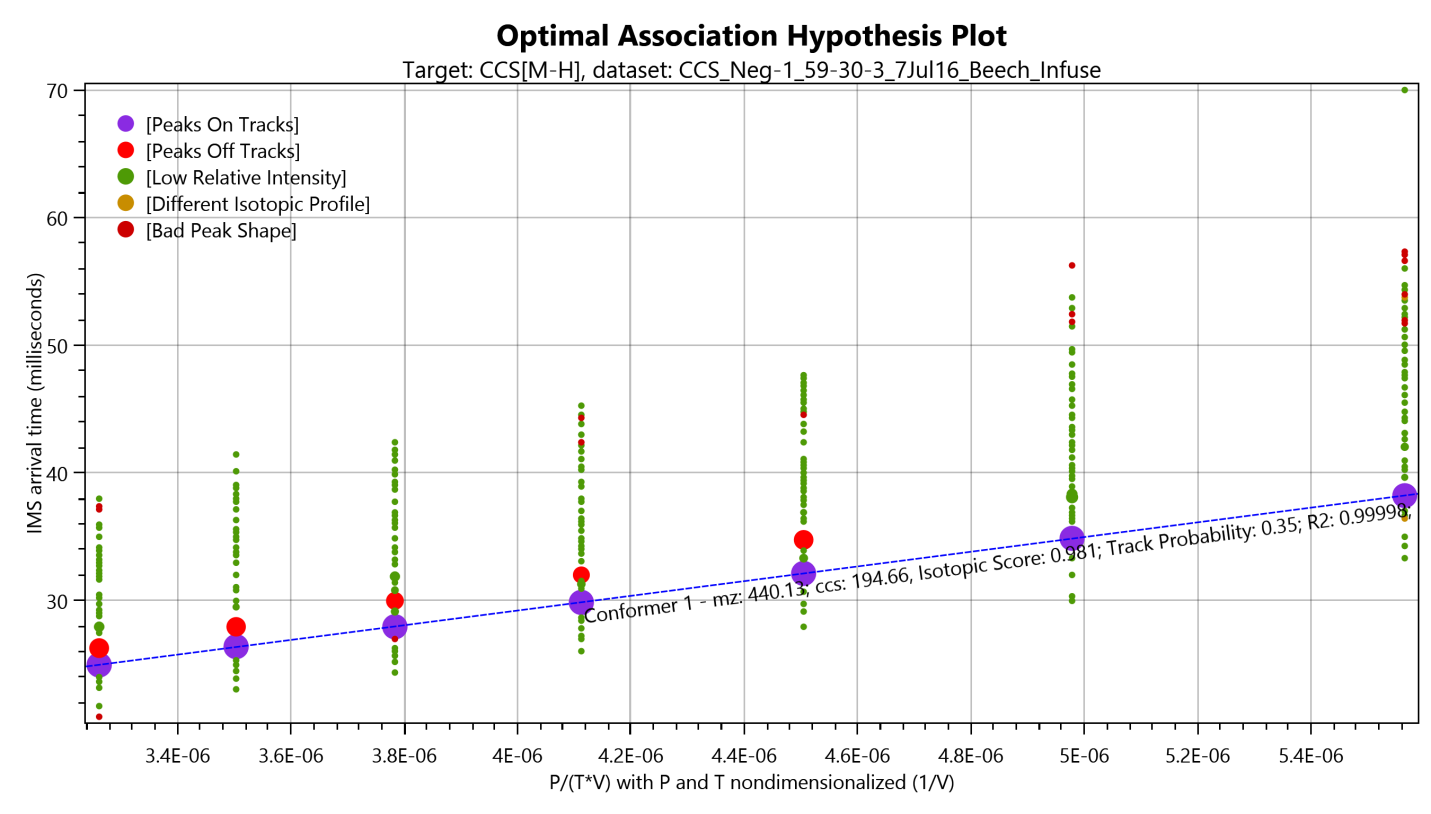
**

**D-Tryptophan [M-H]
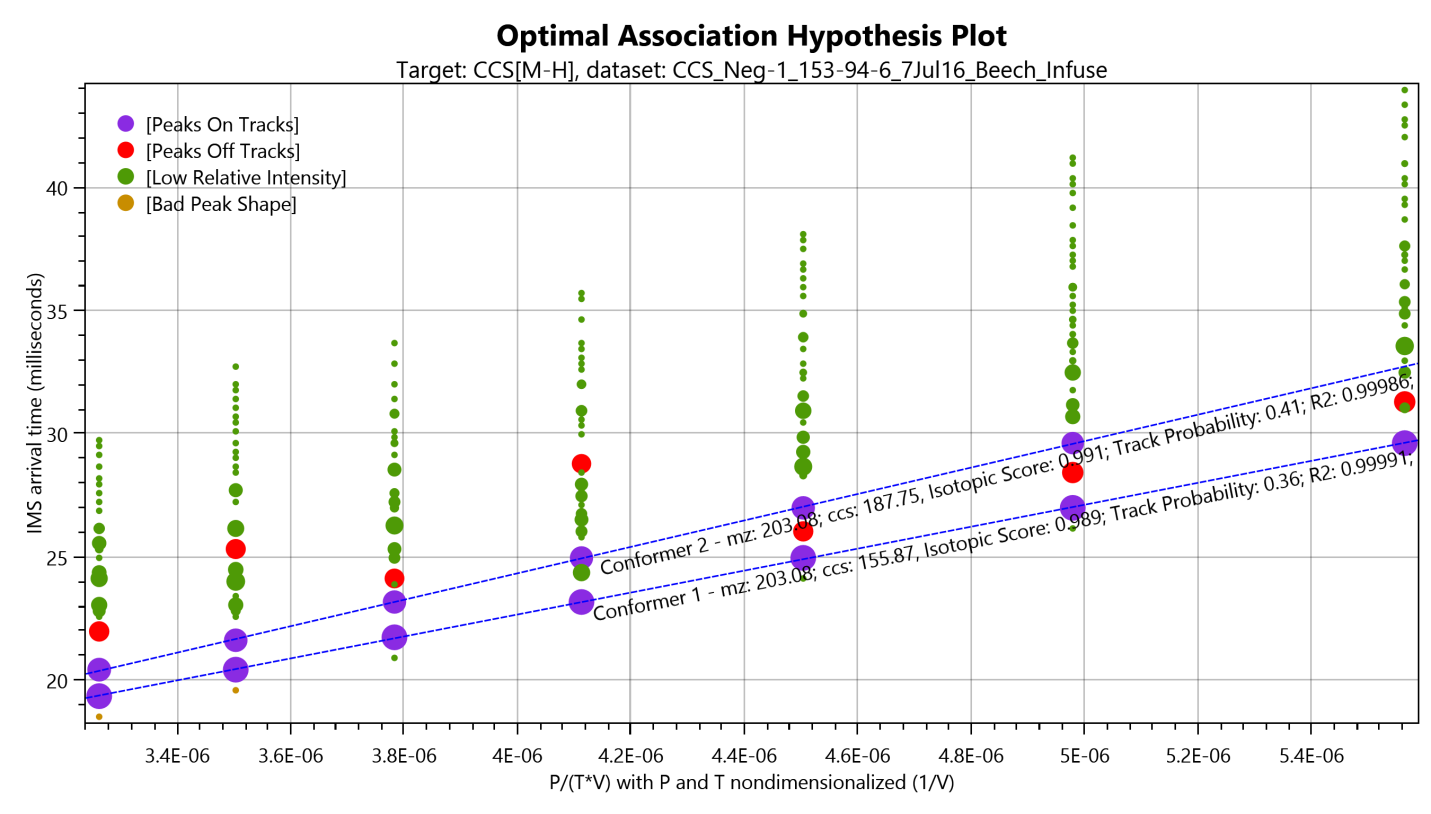
NAD [M-H]
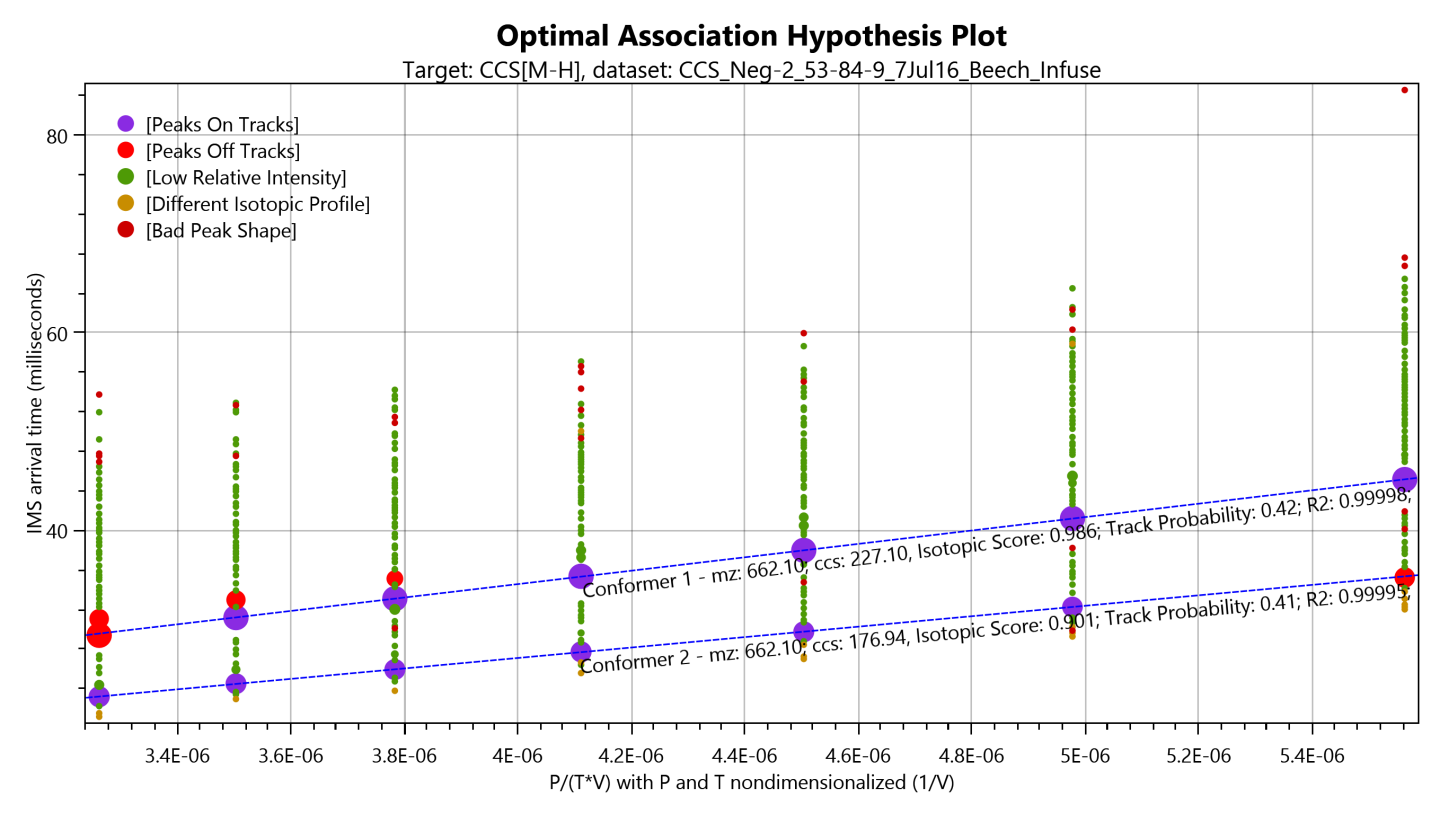
**

**Sucrose [M-H]
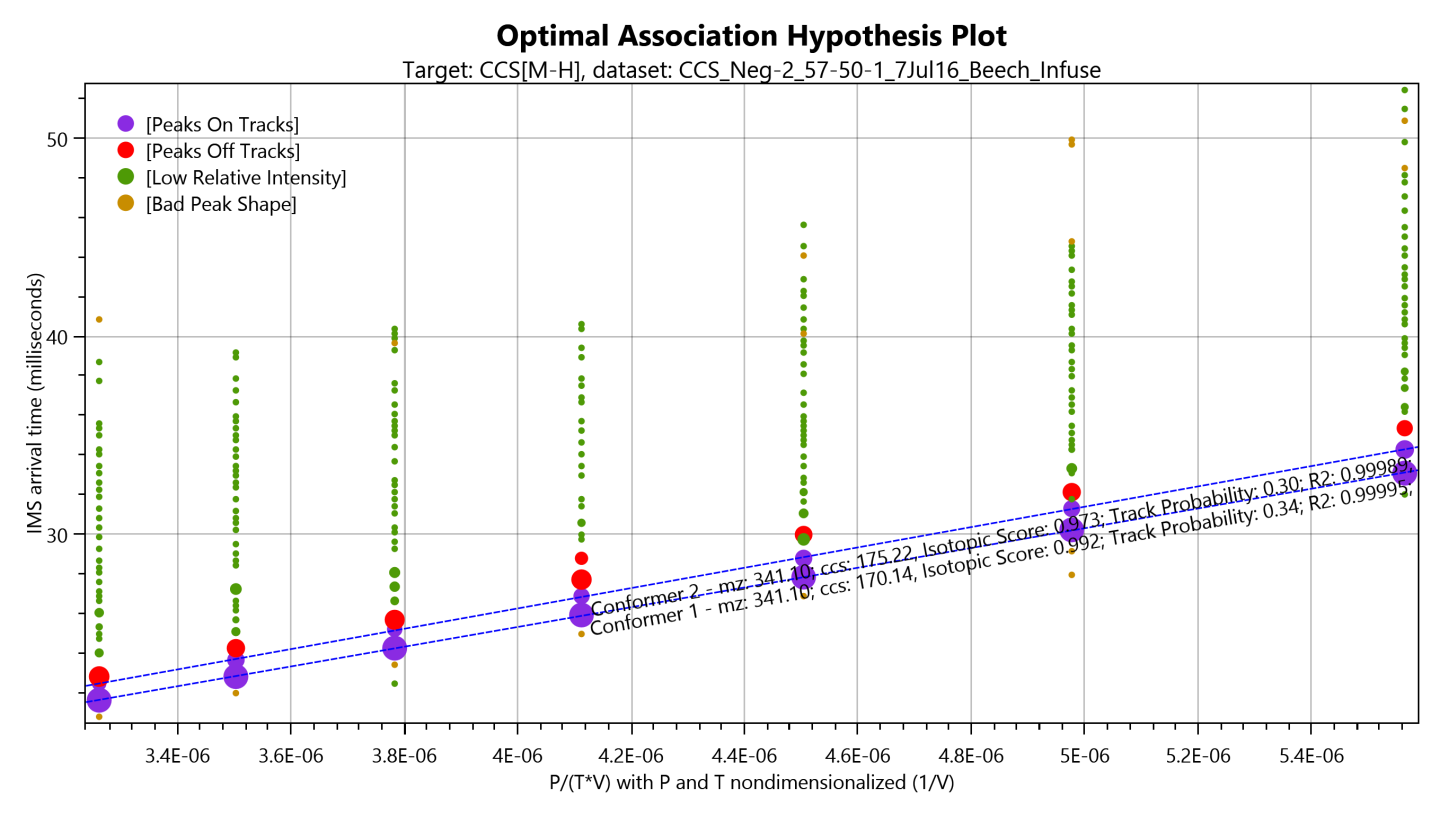
(-)-Epinephrine [M-H]
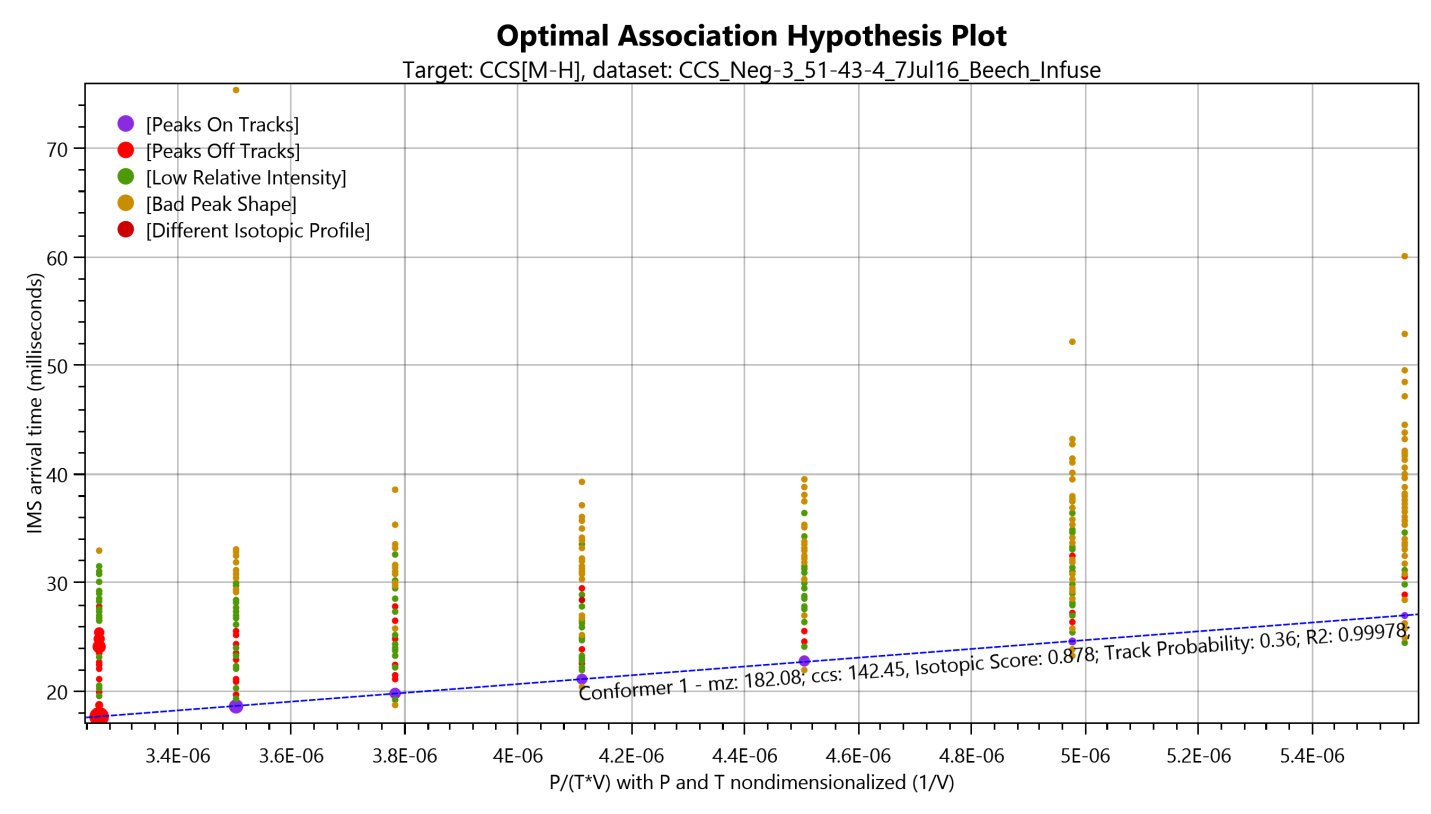
**

**Adenosine [M-H]
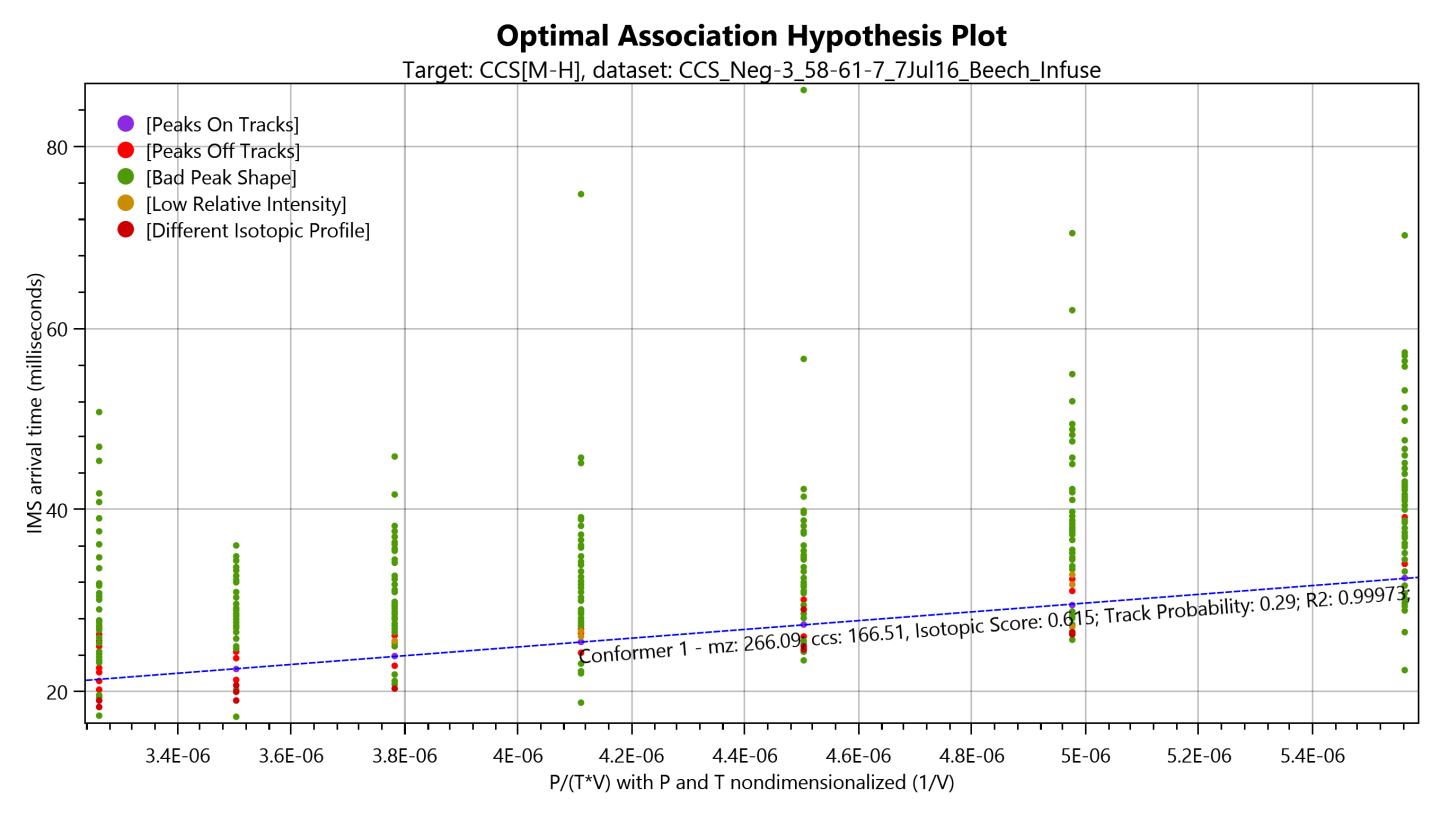
D-Glucosamine 6-phosphate [M-H]
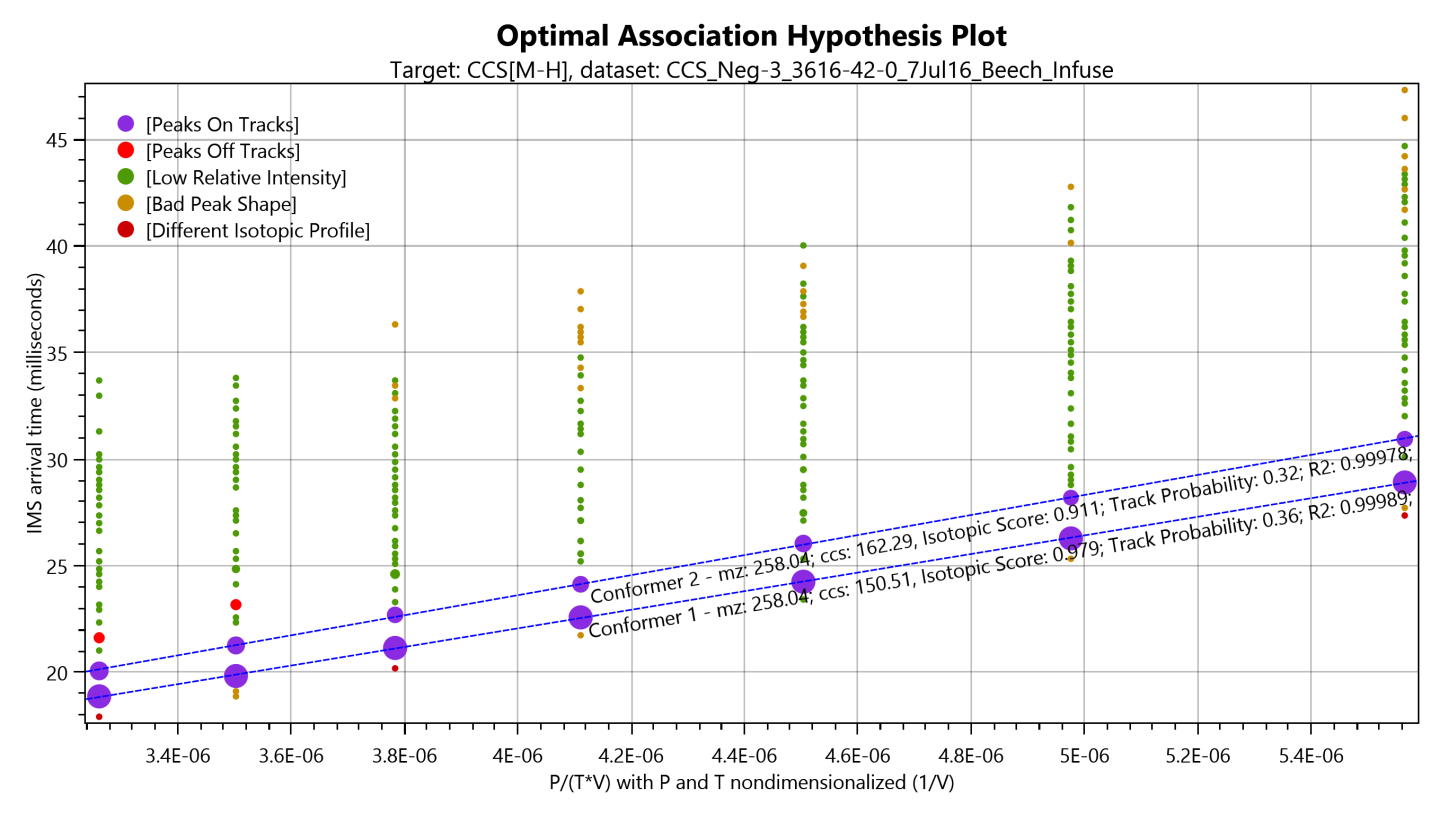
**

**D-Fructose 1,6-diphosphate
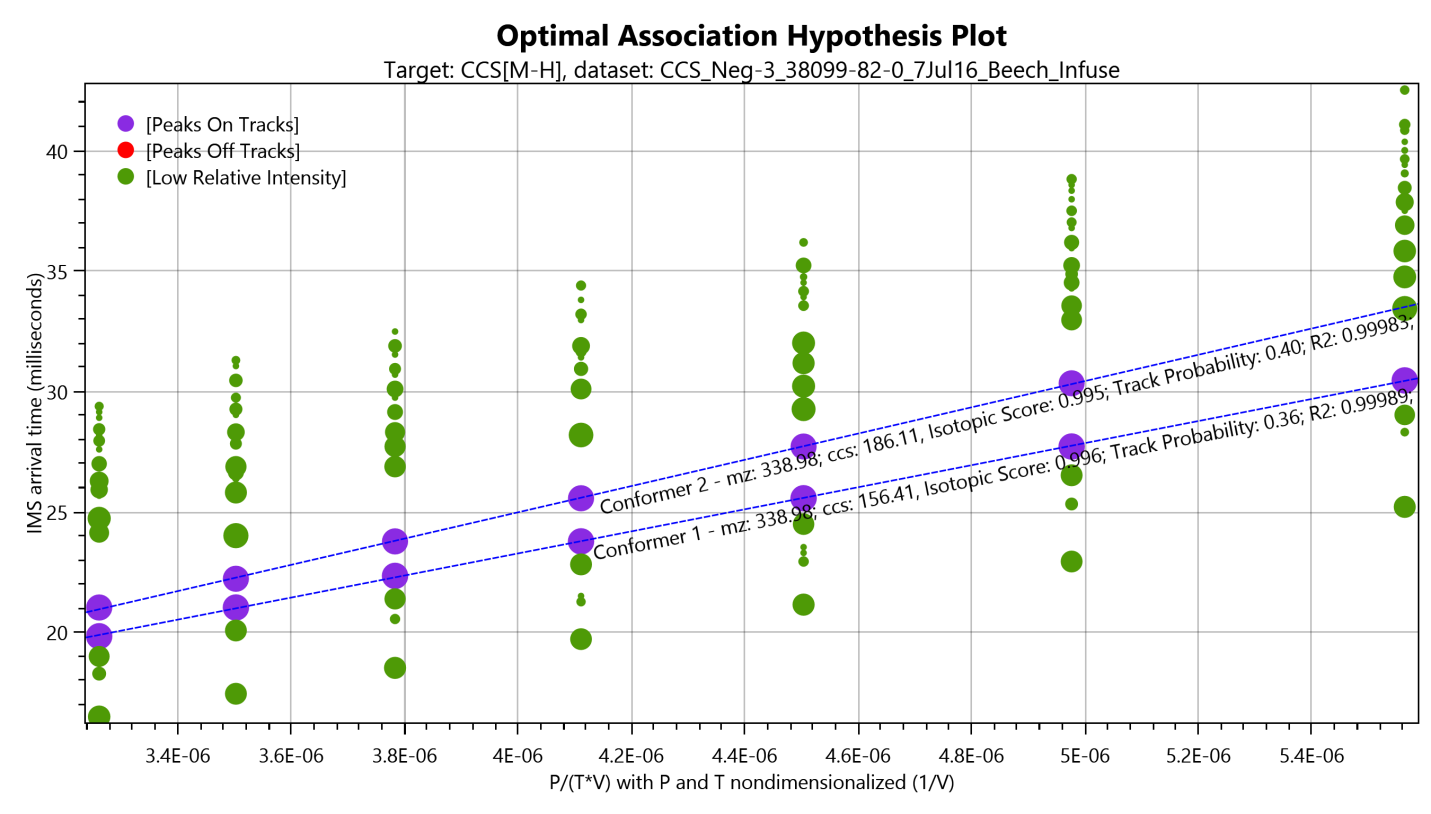
UDP-Galactose [M-H]
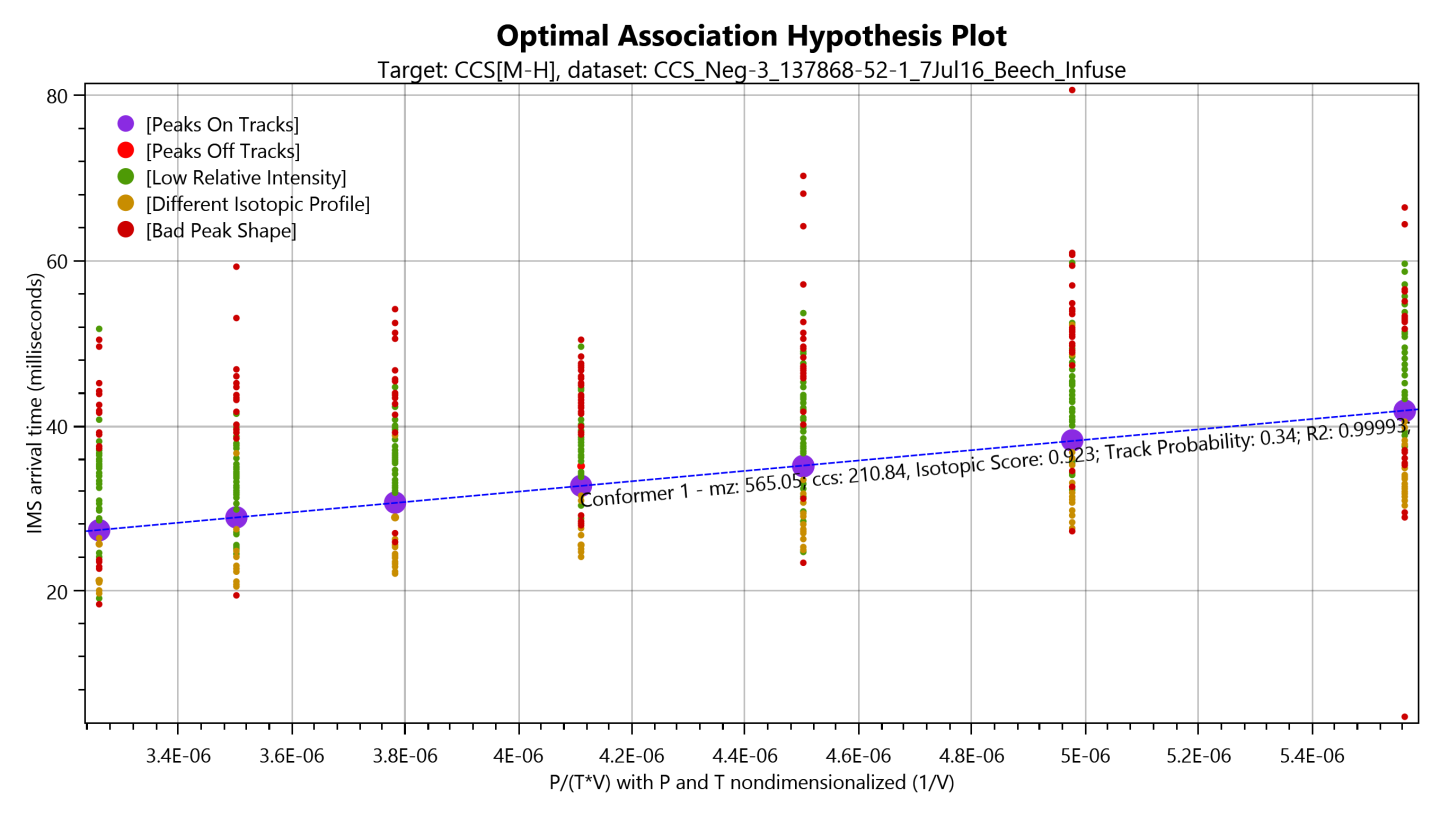
**
